# Supplementary material for: ACCEPTANCE: protocol for a feasibility study of a multicomponent physical activity intervention following treatment for cervical cancer
Source: BMJ Open. 2022 Jan 3;12(1):e048203. doi: 10.1136/bmjopen-2020-048203 (PMC8724712; doi:10.1136/bmjopen-2020-048203)
Supplement: Author's manuscript [file bmjopen-2020-048203.draft_revisions.pdf]

BMJ Open is committed to open peer review. As part of this commitment we make the peer review history of every article we publish publicly available.

When an article is published we post the peer reviewers' comments and the authors' responses online. We also post the versions of the paper that were used during peer review. These are the versions that the peer review comments apply to.

The versions of the paper that follow are the versions that were submitted during the peer review process. They are not the versions of record or the final published versions. They should not be cited or distributed as the published version of this manuscript.

BMJ Open is an open access journal and the full, final, typeset and author-corrected version of record of the manuscript is available on our site with no access controls, subscription charges or pay-per-view fees (http://bmjopen.bmj.com).

If you have any questions on BMJ Open's open peer review process please email info.bmjopen@bmj.com

BMJ Open

ACCEPTANCE: Protocol for a feasibility study of a multicomponent physical activity intervention following treatment for cervical cancer

Journal:	*BMJ Open*
Manuscript ID	bmjopen-2020-048203
Article Type:	Protocol
Date Submitted by the Author:	22-Dec-2020

Complete List of Authors:	Millet, Nessa; Loughborough University School of Sport Exercise and Health Sciences<br>McDermott, Hilary J; Loughborough University School of Sport Exercise and Health Sciences<br>Munir, Fehmidah; Loughborough University School of Sport Exercise and Health Sciences, School of Sport, Exercise and Health Sciences<br>Edwardson, Charlotte; University of Leicester Diabetes Research Centre; NIHR Leicester Biomedical Research Centre<br>Moss, Esther; Leicester Cancer Research Centre, Leicester Cancer Research Centre

Keywords:	Gynaecological oncology < GYNAECOLOGY, PUBLIC HEALTH, SOCIAL MEDICINE

SCHOLARONE™
Manuscripts

TITLE PAGE

Title: ACCEPTANCE: Protocol for a feasibility study of a multicomponent physical activity intervention following treatment for cervical cancer

Corresponding author: Ms Nessa Millet

Postal address: The School of Sport, Exercise and Health Sciences, Sir John Beckwith Centre for Sport, Loughborough University, Epinal Way, Loughborough, Leicestershire, Le11 3TU

Email: N.millet@lboro.ac.uk

Authors: Nessa Millet[1], Hilary J McDermott[1], Fehmidah Munir[1], Charlotte L Edwardson[2,3] Esther L Moss[4]

[1]School of Sport, Exercise and Health Sciences, National Centre for Sport and Exercise Medicine, Loughborough University, Loughborough, UK

[2]Diabetes Research Centre, University of Leicester, Leicester, UK

[3]NIHR Leicester Biomedical Research Centre, Leicester, UK

[4]Leicester Cancer Research Centre, University of Leicester, Leicester, UK

For peer review only

Word count: 2,924

Key words: Gynaecological Oncology, Physical Activity, Quality of Life

ABSTRACT

Introduction: Cervical cancer treatment can have life changing sequelae and is associated with poor short- and long-term quality of life. Physical activity (PA) is known to improve health outcomes and quality of life for cancer survivors, both physically and psychologically. To date, no interventions to increase PA following cervical cancer have been evaluated. Therefore, the purpose of this study is to explore the feasibility and acceptability of conducting and evaluating a PA intervention following treatment for cervical cancer.

Methods and analysis: The design is a pre and post study. Thirty participants aged between 18- 60 years from the Midlands region, UK, who have completed primary treatment for cervical cancer at least 6- months prior recruitment and do not meet the national PA guidelines will be recruited. Identification of potential participants will take place at the University Hospitals of Leicester National Health Service (NHS) Trust. Participants will receive an intervention focused on increasing PA through the provision of education, action planning, goal setting, problem solving and self-monitoring of PA behaviour, particularly steps per day. Device assessed PA and questionnaires will be completed at baseline, week-6, week-12 and week-24. Feasibility will be assessed in terms of recruitment, retention, attrition, completion of measures and intervention compliance. The process evaluation will explore the experiences and acceptability of the intervention components and evaluation measures.

Ethics and dissemination: Ethical approval has been granted by the West of Scotland Research Ethics Committee 1 for this study. Results will be disseminated via peer-reviewed publications and international conferences and will inform intervention refinement for the design of a definitive pilot trial.

Registration details: ISRCTN16349793, Registered 30/09/2020.

For peer review only

ARTICLE SUMMARY

- The integration of behaviour change theory and insight from the target population to inform the development of practical strategies ensures an appropriate and user-friendly intervention.
- A mixed methods process evaluation will provide a great depth of understanding relating to the experiences and acceptability of the intervention and will enhance subsequent refinement where necessary.
- As this is a feasibility study, the analysis will not report statistically powered differences in outcomes (e.g. physical activity).

For peer review only

INTRODUCTION

Advancements in the clinical management of cervical cancer have resulted in improved survival,[1] and as a result have led to a change in focus to treatment-related sequelae and survivorship issues, in order to reduce iatrogenic morbidity and to improve patient's long-term health. Although many patients diagnosed with a cervical cancer have early stage disease (stage IA), which can be successfully treated with local excision, this is often not the case, with 64% of women having at least stage IB disease at diagnosis.[2] As a result, many patients will need to undergo radical surgery and/or chemoradiotherapy, that consequentially leads to the loss of fertility and an early menopause.[3]

The long-term effects of radiotherapy on the vagina,[4] bladder,[5] and bowel can occur in up to 30 to 40% of patients and can not only result in distressing symptoms, urinary/fecal incontinence and tenesmus, but can lead to permanent physical changes, vaginal shortening/stenosis and reduced clitoral sensitivity, thereby physically limiting the ability to have and enjoy intercourse. Chronic fatigue and pelvic insufficiency fractures are also frequently reported following radiotherapy treatment for cervical cancer, the symptoms of which may not diminish with time,[6] and can have a negative impact of daily functions and social quality of life.[7]

There is also substantial evidence of the devastating psychological consequences of a cervical cancer diagnosis, with high proportion of patients experiencing long-term psychological distress following their diagnosis,[8] which can impact on their social, work and functional well-being. Younger patients and those who receive chemoradiotherapy are reportedly more greatly affected,[9] with lower levels of self- confidence and a more negative body image compared to those who undergo surgery alone.[10] Hence, a greater focus on cervical cancer

For peer review only

survivorship is warranted to support patients who are living alongside the physical, psychological and social impacts of cervical cancer treatments.

Identifying and implementing ways to manage treatment side-effects can be challenging, given the individualistic and complex nature of the survivorship experience,[11] and concerns have been raised regarding the knowledge provision of treatment-related morbidity and clinical support services.[12] PA has been shown to be a safe avenue to improve quality of life following cancer treatment,[13] with qualitative data indicating that PA can specifically improve physical, psychological, social and spiritual domains.[14] However, PA levels are reportedly low and remain low up to three years following treatment for a gynaecological malignancy.[15] In particular, cervical cancer survivors meet recommended PA guidelines less frequently than patients following other cancer types.[16]

To date, there has been little research focus on promoting PA amongst those treated for cervical cancer. This is highlighted by cervical cancer cases being underrepresented in meta-analysis studies of gynaecological cancer.[13] Literature based in the broader context of gynaecological cancer and PA does provide some degree of insight regarding PA preferences and feasibility of different types of PA. Such studies suggest that PA which: incorporates home-based walking,[17–19] and gym activities;[19] is graded and flexible;[18] is combined with a form of support or counselling;[18] includes social interaction with peers,[20] tends to be favourable among survivors of gynaecological cancers. There is growing support for technology-based PA promotion among cancer survivors,[21] which can place less burden on participants, and can be easily tailored to the individual.

To the best of our knowledge, an intervention which promotes PA participation following treatment for cervical cancer has not been evaluated. Therefore, we aim to test the feasibility and acceptability of conducting and evaluating a multi-component intervention programme,

For peer review only

informed by relevant theories of behaviour change, to increase PA levels in those who have undergone treatment for cervical cancer, named ACCEPTANCE (Acceptability in Cervical Cancer of an Exercise-based Programme delivered Through An online Community Environment). This intervention was developed using the intervention mapping protocol.[22]

Aim

The overall aim of this study is to assess the feasibility and acceptability of a PA intervention following treatment for cervical cancer.

Objectives

1. To determine participant recruitment, retention and attrition rates
2. To review descriptive data to determine the feasibility of the inclusion criteria
3. To determine compliance and completeness of evaluation measures at all time points
4. To report the feasibility of delivering the intervention and evaluation measures
5. To explore participant compliance with the intervention
6. To obtain views on the acceptability of the intervention and evaluation measures
7. To describe device- assessed PA levels and questionnaire data outcomes of interest

For peer review only

METHODS AND ANALYSIS

Study design

This is a single- arm pre and post study to assess the feasibility and acceptability of conducting and evaluating a PA intervention following treatment for cervical cancer. A process evaluation will also be conducted throughout the study.

Study setting

This study will be conducted through the University Hospitals of Leicester (UHL) NHS Trust, in collaboration with Loughborough University and the University of Leicester.

Patient and Public Involvement (PPI)

PPI input has informed the development of the study since its inception. Insights and feedback gathered from discussions with PPI members enabled assessment of the potential burden on participants and informed the intervention content to ensure that intervention materials were user friendly. Continuation of PPI involvement will inform the intervention delivery and dissemination of results to the wider community as appropriate.

Participants and recruitment

Participants between the ages of 18 and 60 years who have undergone treatment for cervical cancer at least 6 months previously with curative intent (either surgery, chemoradiotherapy or both modalities), who do not meet the national PA guidelines (at least 150 minutes of moderate to vigorous intensity PA per week), who can gain permission from their gynaecologist/ oncologist to take part and are fluent in English will be recruited. Exclusion criteria are benign or pre-malignant (cervical interepithelial neoplasia) disease of the cervix; clinical/radiological evidence of disseminated malignancy; pregnancy or breastfeeding; performance status ≥3; co-

For peer review only

morbidity that in the opinion of the patient's supervising oncologist that would preclude the patient from meeting the study requirements.

Women will be screened prior to recruitment to identify candidates who are at an appropriate stage in their treatment journey. Potential participants will be approached through their clinical team, recruitment posters will be placed in the gynaecological and oncology outpatient departments at UHL and study details (overview of research, inclusion criteria and relevant contact details) will be posted on social media platforms. Local cervical cancer support groups will also be contacted and given information on the study to raise awareness of the research. Screening to determine potential participant's PA levels will take place via a telephone call with a member of the research team. Consent procedures will take place either virtually via video/ telephone call or in person at UHL.

Sample Size

This study will aim to recruit approximately 30 participants. In line with recommendations from the National Institute for Health Research (NIHR), a sample size of 30 is appropriate to answer the questions posed by a feasibility trial.[23] The feasibility of lifestyle and PA interventions in endometrial and breast cancer survivors have successfully employed similar sample sizes.[24,25]

Intervention

The aim of the multi-component intervention is to increase PA levels of the target population, specifically, individual and group-based walking over 12 consecutive weeks (figure 1). Table 1 provides a description of each intervention component whilst figure 2 outlines the Logic Model. Walking has been identified as a common PA preference among gynaecological cancer survivors, particularly for those who do not meet the recommended PA guidelines.[26] The intervention components are underpinned by social cognitive theory (SCT),[27] and are

For peer review only

informed by the health belief model,[28] and theories of self-regulation.[22] Self-monitoring behaviour is strongly linked with successful PA behaviour change, particularly when combined with at least one other self-regulatory component.[29] The intervention includes education provision, problem solving and goal setting in relation to increasing PA levels after treatment for cervical cancer. Self-monitoring of PA behaviour will be facilitated by providing participants with an intervention diary, fortnightly health coaching and a consumer PA self-monitoring device to receive real time feedback on their activity levels and prompts to be more active and to review goals. Throughout, peer support and group walking amongst participants will be encouraged via a messaging platform which will allow participants to maintain contact and organise group walking sessions. The study co-ordinator will suggest appropriate walk locations convenient to each group.

For peer review only

Table 1. Description of intervention components

**Intervention component**	**Description**
Intervention launch	**Education** (interactive session facilitated by the study co-ordinator, delivered virtually)<br>- What is meant by PA?<br>- What kinds of activities count as PA?<br>- What does moderate intensity PA feel like?<br>- What benefits are associated with PA?<br>- Knowledge of PA recommendations<br>- How many steps per day should you aim for?<br>**Barrier Identification**<br>- Is there anything that might stop you from being physically active?<br>- How can you overcome these challenges?<br>**Goal Setting**<br>- Introduction to SMART goals<br>- Using Fitbit inspire to set goals related to steps and intensity<br>*Each section divided by guided 'stand up and stretch' prompts
Self-monitoring of PA	- The PA monitor will provide participants with feedback on their activity levels via: number of steps taken by participants and the number of minutes that participants spend in a moderate intensity activity each day.
Prompts	- Prompts offered by PA monitor to encourage PA<br>- Prompts to update participant on their progress in reaching their daily goal.
Intervention diary (monitoring of well-being)	**Daily entries**<br>- Input daily step count as shown on Fitbit<br>- Rate mood out of 10<br>**Weekly entries**<br>- Rate the following symptoms on a traffic light system: energy levels, anxiety levels, body confidence, physical pain, bladder issues.
Health coaching	- Revision of previous goal(s) and the outcome (successful/ unsuccessful); attribute reasons for this outcome<br>- Identification of challenges and facilitators regarding previous goal<br>- Revision of steps, intensity and well-being over the previous two weeks<br>- Facilitated goal setting and identification of potential challenges for the following 2 weeks
Group messaging	- To be used as a platform to schedule group walks (organising a time and a place) with the aim of one group walk per week. Standardised communication prompts to be offered by the study co-ordinator.
Online community	- Participants will be encouraged to utilise the study specific community on the PA monitor application, accessed via smart phone where personal PA insights and statistics can be shared with other participants.

Measures

All PA and health measures will be assessed at baseline, week-12 and 3- months after completion (week-24), whilst those measures expected to change, based on the literature, will be taken at week- 6 (table 2).

Table 2. PA and health outcomes, outcome measures and measurement time points

**Measures**	**Assessment tool**	**Baseline**	**Week 6**	**Week 12**	**Week 24**
Device- assessed PA	Accelerometer	x	x	x	x
Menopausal Symptoms	MRS	x	x	x	x
Quality of life	EORTC-QLQ-30	x		x	x
Depression and Anxiety	HADS	x		x	x
Fatigue	MSFI-SF	x		x	x
Walking Self- efficacy	Self- efficacy for Walking scale	x		x	x
Motivation for Exercise	BREQ-3	x		x	x
Enjoyment of PA	PACES	x		x	x

PA and health measures

An accelerometer (GENEActiv, Activinsights Ltd) and sleep log will be administered at all evaluation time points to measure PA. The accelerometer will be worn by participants on their non-dominant wrist 24/7 for up to 8 days at each time point.

Participants' belief in their ability to successfully complete incremental 5 to 50- minute periods of walking at a moderate/ brisk pace will be assessed via the self-efficacy for walking scale [30]. Motivation for PA will be assessed using the behavioural regulation in exercise questionnaire (BREQ-3).[31] This is a 23- item scale which assesses motivation on a self-determination continuum. Enjoyment of PA will also be assessed via the original 18- item PACES scale.[32]

In relation to well- being, menopausal symptoms, such as hot flushes and sweating will be investigated using the menopausal rating Scale (MRS).[33] Quality of life will be assessed using the EORTC QLQ- 30, which is commonly used in gynaecological cancer populations. It includes 5 functional scales, 3 symptom scales, a global health scale and a quality of life scale. The Hospital Anxiety and Depression scale (HADS),[34] will be administered to measure symptoms of anxiety and depression. Fatigue will be assessed using the Multi- dimensional fatigue symptom inventory (MFSI-SF),[35] a measure which has been previously used to assess fatigue in a gynaecological cancer population.[17]

Evaluation related feasibility measures

Participant recruitment rates will be monitored by recording the number of identified potential participants, the number of those who express an interest in taking part, the number of those who are eligible and not eligible along with the reasons for ineligibility and the number who consent to participate. Retention and attrition rates will be monitored by recording the number of participants who withdraw from the study and the number who do not attend follow-up.

For peer review only

Where possible, reasons for withdrawal will also be reported. The completion rates of the PA and health measures at each time point will be summarised.

Process evaluation

The process evaluation will be conducted to explore participant experiences of the study and intervention as well as compliance with the intervention components, to identify facilitators and barriers to increasing PA and any suggestions for improvement and refinement. Compliance with intervention components will be reviewed throughout the intervention. Data will be presented on launch attendance rates, the number of diary entries completed, number of daily step counts recorded to reflect compliance of wearing the PA monitor, number of health coaching sessions attended, the number of participants who post on the online forum and engage in group messaging, the number of participants who take part in group walks and the frequency of these.

Questionnaires will be administered to understand participant's experience of attending the intervention launch, of using the PA monitor and the group messaging. A semi- structured interview will also be conducted to further explore perceptions of the intervention and the evaluation measures, perceived benefits of participation, maintenance of the intervention components and PA levels. Interview data will be analysed using Template Analysis,[36] employing a deductive coding framework to inform study refinement. To ensure a balanced insight and that subsequent meaningful refinement can be implemented, some transcripts will be coded by an independent researcher who did not contribute to the intervention design.

A researcher log will be completed by the study co-ordinator where a record of interactions with participants (e.g. email conversations/ telephone conversations) will be kept throughout the duration of the study. Feedback will be broadly divided into four categories: evaluation of intervention components; experience of evaluation measures; alterations to future intervention implementation; barriers to PA and reasons for discontinuation (where relevant).

For peer review only

Data collection

The accelerometer and the quantitative measures will be posted to participants. Process evaluation questionnaires will be completed online throughout the intervention. The evaluation interview will be offered after the week-24 time point to all participants (including those who have withdrawn) either in person or via video call.

Statistical Analysis

As this is a feasibility study, main analyses will utilise descriptive statistics. Data will be analysed using *IBM SPSS statistics 25*.

The feasibility of inclusion criteria

we have suggested that if an eligibility criterion accounts for 40% of ineligibility, it will be investigated further.

The feasibility of study recruitment and retention

A priori, we have defined a recruitment success rate as recruitment of 30 participants in the first 3- months or that 75% of those identified are recruited, and a successful retention rate at week-24 as 70%.

The feasibility of and compliance with intervention components

Attendance rates at the intervention launch, engagement with the online forum, frequency of wearing the PA monitor, frequency of group walking sessions, compliance with diary completion and attendance rates at health coaching sessions will all be summarised.

The feasibility of the evaluation measures

Compliance with evaluation measures will be deemed successful if no more than 20% of participants fail to provide questionnaire and accelerometer data at both baseline and week-24 follow-up.

For peer review only

Physical activity data and questionnaire outcomes

Data from the accelerometer will be cleaned and processed in GGIR using *R programming* and analysed using *IBM SPSS statistics 25*. Means and standard deviation (SD) will be used to describe the PA from the accelerometer at each evaluation time point. Constructs measured via questionnaire will be analysed by standard scoring procedures. Questionnaire outcomes of interest will be reported on means (or medians where necessary) and SDs.

Ethics and dissemination

Ethical approval has been granted by the West of Scotland Research Ethics Committee 1 for this study. Results will primarily be used to refine intervention components and to inform a larger full-scale trial, if appropriate. Results from the study will be shared in peer reviewed journals, presented at international conferences and will be shared with the University Hospitals of Leicester and Leicester Hospital's charity.

For peer review only

DISCUSSION

This article describes the protocol for a study testing the feasibility and acceptability of conducting and evaluating of a multi- component PA intervention following treatment for cervical cancer. Regular PA has been shown to have physical and psychological benefits and to be safe after cancer .[37] PA can also alleviate some of the symptoms experienced as a result of cancer treatment .[17] PA levels appear to be lower following cervical cancer and this may be partly due to the lack of a specific intervention that takes into consideration the particular side-effects that can occur, in particular pelvic pain and incontinence, that may not be such an issue in other cancer types.

The study protocol details a 12-week intervention programme to promote walking and PA, with evaluation measures taken at baseline, 6- weeks, 12 weeks and 24 weeks. The process evaluation is a mix of questionnaires and a qualitative interview. The intervention was designed using the intervention mapping protocol.[22] Development followed a systematic 6-step framework which prioritises input from the target population as well as integrating behaviour change theories (BCTs) and constructs to underpin change within the intervention, informed by relevant literature.[38] Interventions underpinned by relevant BCTs have been found to be more successful in implementing change and in identifying more specific evaluation criteria.[37]

The current PA guidelines offered by the Government Chief Medical Officers do not specify recommendations for cervical cancer survivors.[39] However, using a development framework that prioritises insights and experiences from the target population is a great strength of this intervention, as it allowed direct and effective tailoring of the intervention components despite specific recommendations not being available. Hence, the aerobic PA recommendations were deemed an appropriate target for women treated for cervical cancer. Additionally, the criterion to include women who are at least 6- months post treatment was also

For peer review only

a decision informed by the need's assessment, with the time between 3 and 9 months post treatment being identified as critical for intervention and for positive change.[40] A limitation of this protocol is that it does not incorporate strength training, and therefore does not reflect the current PA guidelines for after cancer. However, as PA levels are generally low following cervical cancer treatment, it seemed appropriate to promote one PA modality which was realistically achievable (i.e. walking) to enhance adherence to the intervention.

This study will add to the current debate regarding the role of technology in promoting PA after cancer. The intervention utilises a PA monitor and its associated online community and a messaging platform. Previous research supports the use of such e-health technologies in combination with health coaching over a shorter duration (4-week programme).[41] However, it has been suggested that further investigation is needed to test the acceptability of such technologies in more diverse populations,[42] to explore optimal intervention length and to question the maintenance of PA behaviour change.[41] Although a limitation of this protocol is that statistically relevant changes in PA between time points will not be reported, trends in PA levels will provide data inferring the feasibility of promoting and maintaining PA behaviours. The process evaluation will also provide valuable insight regarding the acceptability of the components.

For peer review only

CONCLUSION

We described the protocol of a study which tests the feasibility and acceptability of delivering and evaluating a multi- component physical activity intervention for women following treatment for cervical cancer. A process evaluation plan will provide insight into whether the intervention components and evaluation measures are accepted by participants. Findings from the study will inform intervention refinement in preparation for a full-scale pilot trial.

For peer review only

Contributorship statement

All authors contributed to the study conception and design and protocol preparation. The first draft of the manuscript was written by NM and EM. CE, FM and HJM significantly revised the manuscript. All authors read and approved the final manuscript.

Competing Interest statement

ELM has received research grants from Intuitive Surgical and Hope Against Cancer for unrelated work. CLE has received lecture fees for GSK and is on the clinical advisory board for Inivata.

Funding Statement

This research is supported by the National Institute for Health Research (NIHR) Leicester Biomedical Research Centre, which is a partnership between University Hospitals of Leicester NHS Trust, Loughborough University and the University of Leicester, UK. Support. Funding was provided by the Economic, Social and Research Council (ES/P000711/1), Loughborough university (ES/P000711/1), and the Leicester Hospital's charity, University Hospitals of Leicester (Q843).

Acknowledgements

The authors are very grateful to the members of the Patient and Public Involvement group for their contributions and insights throughout the intervention development stages.

REFERENCES

1 Cancer Research UK. Cervical cancer statistics. 2017. https://www.cancerresearchuk.org/health-professional/cancer-statistics/statistics-by-cancer-type/cervical-cancer#heading-Three (Date accessed: OCT 2020)

2 Public Health England. Cervical cancer screening: invasive cervical cancer audit 2013 to 2016. 2019.https://www.gov.uk/government/publications/cervical-screening-invasive-cervical-cancer-audit-2013-to-2016/executive-summary#staging-information (Date accessed: OCT 2020)

3 Moss EL, Taneja S, Munir F, *et al.* Iatrogenic Menopause After Treatment for Cervical Cancer. *Clin Oncol* 2016;**28**:766–75. doi:10.1016/j.clon.2016.08.016

4 Bergmark K, Åvall-Lundqvist E, Dickman PW, *et al.* Patient-rating of distressful symptoms after treatment for early cervical cancer. *Acta Obstet Gynecol Scand* Published Online First: 2002. doi:10.1034/j.1600-0412.2002.810512.x

5 Pisarska M, Sajdak S. Lower urinary tract function after postoperative radiotherapy in the treatment of cervical cancer. *Eur J Gynaecol Oncol* 2003.

6 Elghamrawi KA, Haggag MH, Habib EE. Treatment complications among long-term survivors of cervical cancer: Treated by surgery or radiotherapy. Oncol. Rev. 2007. doi:10.1007/s12156-011-0093-0

7 Schover LR, Fife M, Gershenson DM. Sexual dysfunction and treatment for early stage cervical cancer. *Cancer* Published Online First: 1989. doi:10.1002/1097-0142(19890101)63:1<204::AID-CNCR2820630133>3.0.CO;2-U

8 Le Borgne G, Mercier M, Woronoff AS, *et al.* Quality of life in long-term cervical cancer survivors: A population-based study. *Gynecol Oncol* 2013;**129**:222–8. doi:10.1016/j.ygyno.2012.12.033

9 Derks M, Van Lonkhuijzen LRCW, Bakker RM, *et al.* Long-Term Morbidity and Quality of Life in Cervical Cancer Survivors: A Multicenter Comparison between Surgery and Radiotherapy as Primary Treatment. *Int J Gynecol Cancer* Published Online First: 2017. doi:10.1097/IGC.0000000000000880

10 Kirchhoff AC, Yi J, Wright J, *et al.* Marriage and divorce among young adult cancer survivors. *J Cancer Surviv* Published Online First: 2012. doi:10.1007/s11764-012-0238-6

11 Sekse RJT, Raaheim M, Blaaka G, *et al.* Life beyond cancer: Women's experiences 5-years after treatment for gynaecological cancer. *Scand J Caring Sci* 2010;**24**:799–807. doi:10.1111/j.1471-6712.2010.00778.x

12 Ciudad P, Manrique OJ, Agko M, *et al.* Reply to comment on: Ileocecal vascularized lymph node transfer for the treatment of extremity lymphedema: A case report. Microsurgery. 2018. doi:10.1002/micr.30336

13 Buffart LM, Kalter J, Sweegers MG, *et al.* Effects and moderators of exercise on quality of life and physical function in patients with cancer: An individual patient data meta-analysis of 34 RCTs. Cancer Treat. Rev. 2017. doi:10.1016/j.ctrv.2016.11.010

For peer review only

14 Burke S, Wurz A, Bradshaw A, *et al.* Physical activity and quality of life in cancer survivors: A meta-synthesis of qualitative research. Cancers (Basel). 2017. doi:10.3390/cancers9050053

15 Fleming S, Jones T, Janda M, *et al.* Physical activity trajectories following gynecological cancer: results from a prospective, longitudinal cohort study. *Int J Gynecol Cancer* Published Online First: 2020. doi:10.1136/ijgc-2020-001543

16 Thraen-Borowski KM, Gennuso KP, Cadmus-Bertram L. Accelerometer-derived physical activity and sedentary time by cancer type in the United States. *PLoS One* Published Online First: 2017. doi:10.1371/journal.pone.0182554

17 Donnelly CM, Blaney JM, Lowe-Strong A, *et al.* A randomised controlled trial testing the feasibility and efficacy of a physical activity behavioural change intervention in managing fatigue with gynaecological cancer survivors. *Gynecol Oncol* 2011;**122**:618–24. doi:10.1016/j.ygyno.2011.05.029

18 Donnelly CM, Lowe-Strong A, Rankin JP, *et al.* A focus group study exploring gynecological cancer survivors' experiences and perceptions of participating in a RCT testing the efficacy of a home-based physical activity intervention. *Support Care Cancer* Published Online First: 2013. doi:10.1007/s00520-012-1716-0

19 Brown JC, John GM, Segal S, *et al.* Physical activity and lower limb lymphedema among uterine cancer survivors. *Med Sci Sports Exerc* Published Online First: 2013. doi:10.1249/MSS.0b013e318299afd4

20 Rossi A, Garber CE, Ortiz M, *et al.* Feasibility of a physical activity intervention for obese, socioculturally diverse endometrial cancer survivors. *Gynecol Oncol* Published Online First: 2016. doi:10.1016/j.ygyno.2016.05.034

21 Rossi A, Frechette L, Miller D, *et al.* Acceptability and feasibility of a Fitbit physical activity monitor for endometrial cancer survivors. *Gynecol Oncol* Published Online First: 2018. doi:10.1016/j.ygyno.2018.04.560

22 Bartholomew, Eldredge L Highfield L, Hartman M, Mullen P, *et al.* Using Intervention Mapping to Adapt Evidence-Based Interventions. In: *Planning health promotion programs : an intervention mapping approach*. 2016.

23 Lancaster GA, Dodd S, Williamson PR. Design and analysis of pilot studies: Recommendations for good practice. J. Eval. Clin. Pract. 2004. doi:10.1111/j..2002.384.doc.x

24 Rossi A, Frechette L, Miller D, *et al.* Acceptability and feasibility of a Fitbit physical activity monitor for endometrial cancer survivors. *Gynecol Oncol* 2018;**149**:470–5. doi:10.1016/j.ygyno.2018.04.560

25 Pullen T, Sharp P, Bottorff JL, *et al.* Acceptability and satisfaction of project MOVE: A pragmatic feasibility trial aimed at increasing physical activity in female breast cancer survivors. *Psychooncology* Published Online First: 2018. doi:10.1002/pon.4662

26 Stevinson C, Tonkin K, Capstick V, *et al.* A Population-Based Study of the Determinants of Physical Activity in Ovarian Cancer Survivors. *J Phys Act Heal* 2009;**6**:339–46. doi:10.1123/jpah.6.3.339

27 Bandura A. Social foundations of thought and action : a social cognitive theory / Albert Bandura. *New Jersey Prentice-Hall, 1986* 1986.

For peer review only

28 Janz K, Becker MH. The Health Belief Model : A Decade Later. 1984;:1–47.

29 Michie S, Abraham C, Whittington C, *et al.* Effective Techniques in Healthy Eating and Physical Activity Interventions: A Meta-Regression. *Heal Psychol* Published Online First: 2009. doi:10.1037/a0016136

30 McAuley E, Blissmer B, Katula J, *et al.* Exercise environment, self-efficacy, and affective responses to acute exercise in older adults. *Psychol Heal* Published Online First: 2000. doi:10.1080/08870440008401997

31 Markland D, Tobin V. A modification to the behavioural regulation in exercise questionnaire to include an assessment of amotivation. *J Sport Exerc Psychol* Published Online First: 2004. doi:10.1123/jsep.26.2.191

32 Kendzierski D, DeCarlo KJ. Physical Activity Enjoyment Scale: Two Validation Studies. *J Sport Exerc Psychol* Published Online First: 2016. doi:10.1123/jsep.13.1.50

33 Hauser GA, Huber IC, Keller PJ, *et al.* EVALUATION DER KLIMAKTERISCHEN BESCHWERDEN (MENOPAUSE RATING SCALE [MRS]). Zentralbl. Gynakol. 1994.

34 Zigmond, Anthony S, Snaith, Philip R. The Hospital Anxiety and Depression Scale. *Acta Psychiatr Scand* 1983;**67**:361–70.

35 Stein KD, Jacobsen PB, Blanchard CM, *et al.* Further validation of the multidimensional fatigue symptom inventory-short form. *J Pain Symptom Manage* Published Online First: 2004. doi:10.1016/j.jpainsymman.2003.06.003

36 King N. Template Analysis website. 2014.https://research.hud.ac.uk/research-subjects/human-health/template-analysis/ (Date accessed: NOV 2020).

37 Turner RR, Steed L, Quirk H, *et al.* Interventions for promoting habitual exercise in people living with and beyond cancer. Cochrane Database Syst. Rev. 2018. doi:10.1002/14651858.CD010192.pub3

38 Hirschey R, Bryant AL, Macek C, *et al.* Predicting physical activity among cancer survivors: Meta-analytic path modeling of longitudinal studies. *Heal Psychol* Published Online First: 2020. doi:10.1037/hea0000845

39 GOV.UK. UK Chief Medical Officers' Physical Activity Guidelines. 2019;:66.https://assets.publishing.service.gov.uk/government/uploads/system/uploads/attachment_data/file/832868/uk-chief-medical-officers-physical-activity-guidelines.pdf (Date accessed: NOV 2020)

40 McDonough MH, Sabiston CM, Sedgwick WA, *et al.* Changes in Intrinsic Motivation and Physical Activity among Overweight Women in a 12-Week Dragon Boat Exercise Intervention Study. *Women Sport Phys Act J* Published Online First: 2016. doi:10.1123/wspaj.19.1.33

41 Gell NM, Grover KW, Humble M, *et al.* Efficacy, feasibility, and acceptability of a novel technology-based intervention to support physical activity in cancer survivors. *Support Care Cancer* Published Online First: 2017. doi:10.1007/s00520-016-3523-5

42 Haberlin C, O'Dwyer T, Mockler D, *et al.* The use of eHealth to promote physical activity in cancer survivors: a systematic review. Support. Care Cancer. 2018. doi:10.1007/s00520-018-4305-z

For peer review only

Protocol amendments

Any modifications to the protocol will be approved by the West of Scotland Research Ethics Committee 1 prior to implementation and notified to the health authorities in accordance with local regulations.

Ethical approvals

Ethical approval was given by the West of Scotland Research Ethics Committee (20/WS/0062).

List of Figures

Figure 1. Intervention Programme Flowchart

*Figure 2***.** Logic Model for the ACCEPTANCE trial

For peer review only

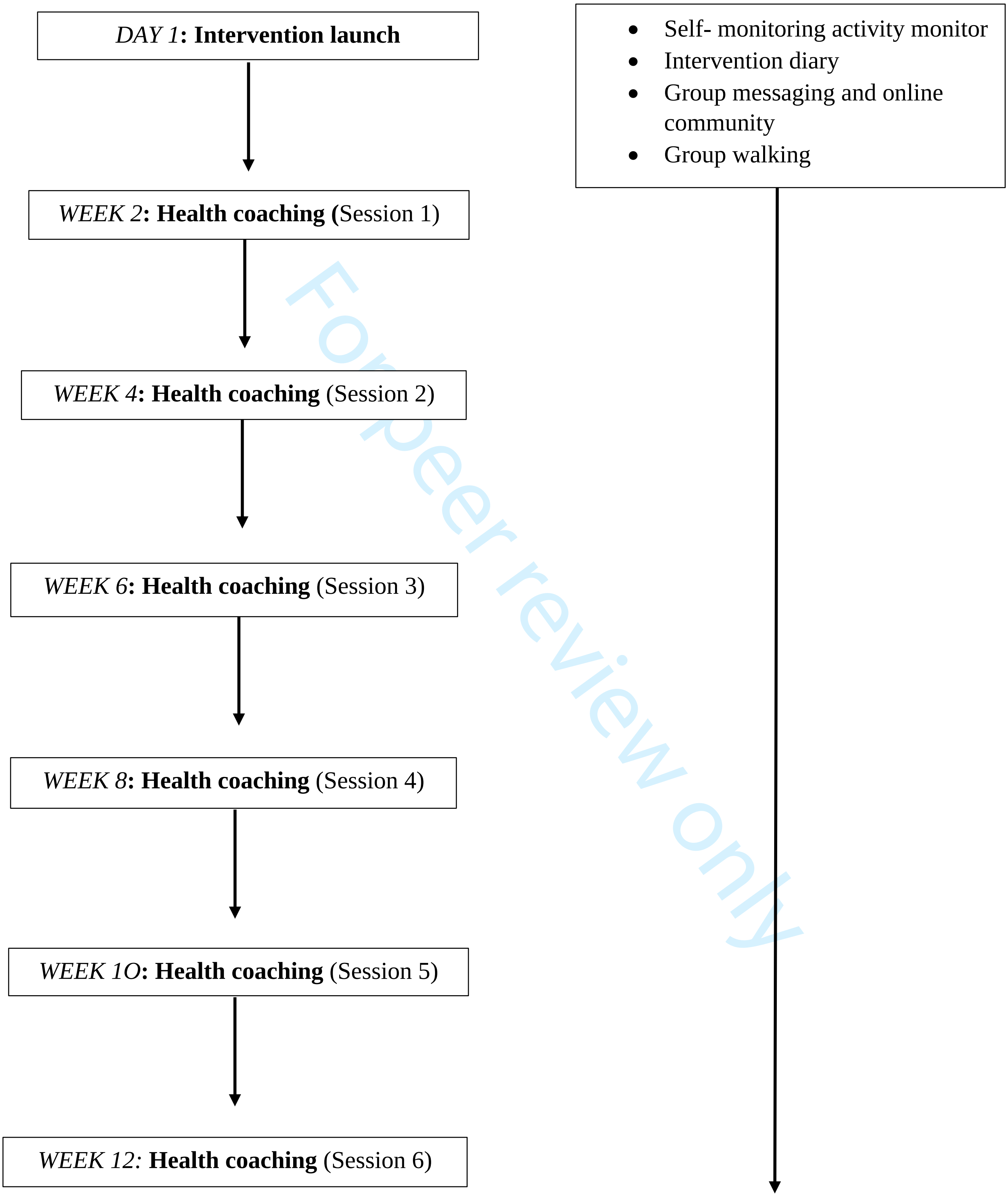

Figure 1. Intervention programme flowchart

For peer review only

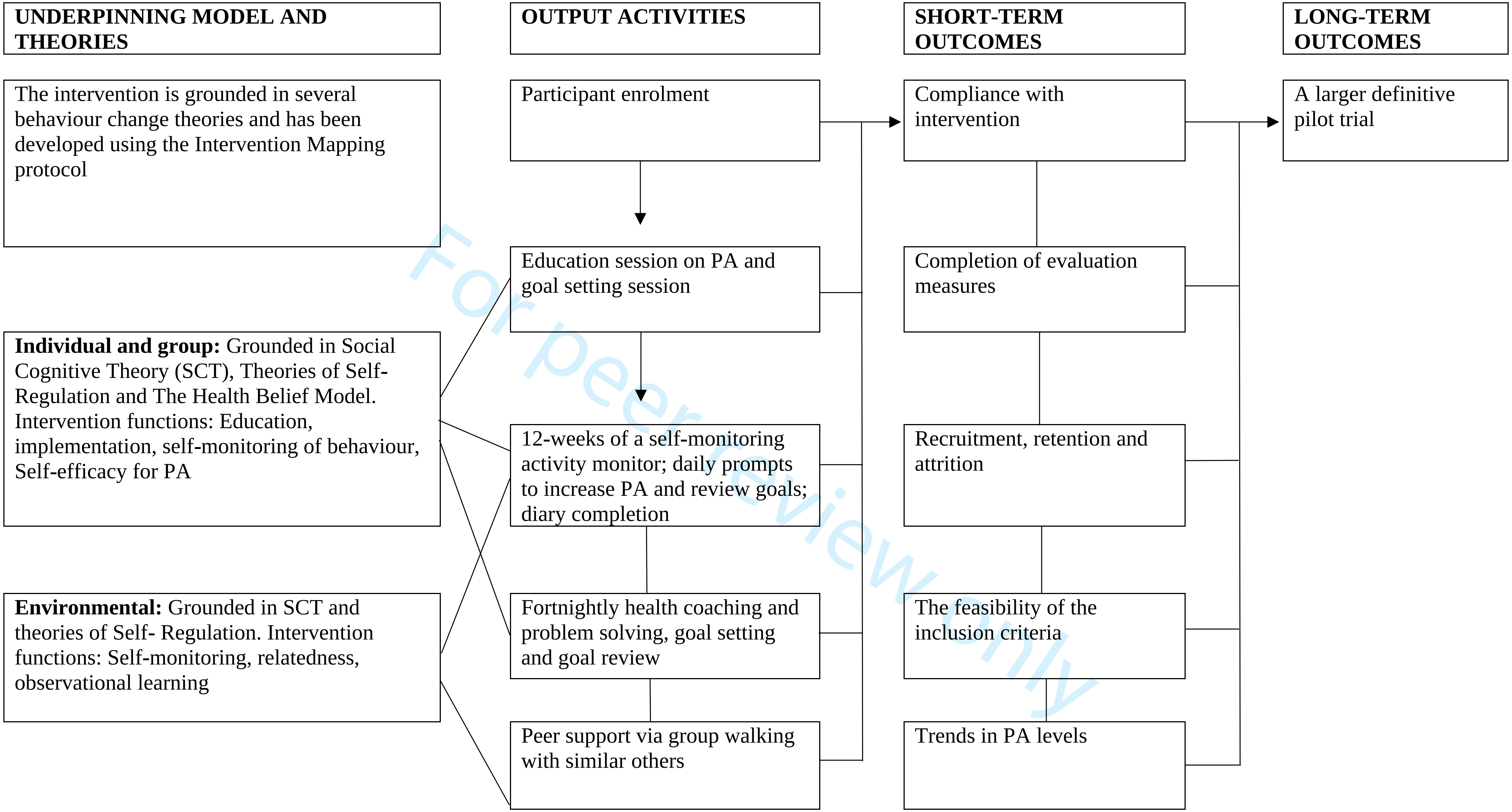

Figure 2. The logic model for the ACCEPTANCE study

For peer review only - http://bmjopen.bmj.com/site/about/guidelines.xhtml

BMJ Open

ACCEPTANCE: Protocol for a feasibility study of a multicomponent physical activity intervention following treatment for cervical cancer

Journal:	*BMJ Open*
Manuscript ID	bmjopen-2020-048203.R1
Article Type:	Protocol
Date Submitted by the Author:	15-Sep-2021

Complete List of Authors:	Millet, Nessa; Loughborough University School of Sport Exercise and Health Sciences<br>McDermott, Hilary J; Loughborough University School of Sport Exercise and Health Sciences<br>Munir, Fehmidah; Loughborough University School of Sport Exercise and Health Sciences, School of Sport, Exercise and Health Sciences<br>Edwardson, Charlotte; University of Leicester Diabetes Research Centre; NIHR Leicester Biomedical Research Centre<br>Moss, Esther; Leicester Cancer Research Centre, Leicester Cancer Research Centre

**Primary Subject Heading**:	Sports and exercise medicine
Secondary Subject Heading:	Oncology, Public health
Keywords:	Gynaecological oncology < GYNAECOLOGY, PUBLIC HEALTH, SOCIAL MEDICINE

SCHOLARONE™
Manuscripts

TITLE PAGE

Title: ACCEPTANCE: Protocol for a feasibility study of a multicomponent physical activity intervention following treatment for cervical cancer

Corresponding author: Dr Esther L Moss

Postal address: Leicester Cancer Research Centre, University of Leicester, Leicester, UK, LE2 7LX. Email: em321@le.ac.uk. TEL: +44 116 2523170 Fax: +44 116 258 8210

Authors: Nessa Millet[1], Hilary J McDermott[1], Fehmidah Munir[1], Charlotte L Edwardson[2,3] Esther L Moss[4]

[1]School of Sport, Exercise and Health Sciences, National Centre for Sport and Exercise Medicine, Loughborough University, Loughborough, UK

[2]Diabetes Research Centre, University of Leicester, Leicester, UK

[3]NIHR Leicester Biomedical Research Centre, Leicester, UK

[4]Leicester Cancer Research Centre, University of Leicester, Leicester, UK

Word count: 3,033

Key words: Gynaecological Oncology, Physical Activity, Quality of Life

For peer review only

For peer review only - http://bmjopen.bmj.com/site/about/guidelines.xhtml

ABSTRACT

Introduction: Cervical cancer treatment can have life changing sequelae and is associated with poor short- and long-term quality of life. Physical activity (PA) refers to any movement that uses energy. PA is known to improve health outcomes and quality of life for cancer survivors, both physically and psychologically. To date, no interventions to increase PA following cervical cancer have been evaluated. This study aims to (1) determine the feasibility of conducting a PA intervention after cervical cancer and (2) to explore the acceptability of the programme and evaluation measures.

Methods and analysis: The design is a pre and post study. Thirty participants aged between 18- 60 years from the Midlands region, United Kingdom, who have completed primary treatment for cervical cancer at least 6- months prior recruitment and do not meet the national PA guidelines will be recruited. Identification of potential participants will take place at the University Hospitals of Leicester National Health Service (NHS) Trust. Participants will receive an intervention focused on increasing PA through the provision of education, action planning, goal setting, problem solving and self-monitoring of PA behaviour, particularly steps per day. Device assessed PA and questionnaires will be completed at baseline, week-6, week-12 and week-24. Feasibility will be assessed in terms of recruitment, retention, attrition, completion of measures and intervention compliance for which, specific feasibility criteria have been established. The process evaluation will explore the experiences and acceptability of the intervention components and evaluation measures.

Ethics and dissemination: Ethical approval has been granted by the West of Scotland Research Ethics Committee 1 for this study. Results will inform intervention refinement for the design of a definitive pilot trial. These results will be disseminated via peer-reviewed publications and international conferences whilst input from a patient and public involvement (PPI) group will inform effective ways to circulate results amongst the wider community.

For peer review only - http://bmjopen.bmj.com/site/about/guidelines.xhtml

Registration details: ISRCTN16349793, Registered 30/09/2020.

ARTICLE SUMMARY

- The integration of behaviour change theory and insight from the target population to inform the development of practical strategies ensures an appropriate and user-friendly intervention.
- A mixed methods process evaluation will provide a great depth of understanding relating to the experiences and acceptability of the intervention and will enhance subsequent refinement where necessary.
- As this is a feasibility study, the analysis will not report statistically powered differences in outcomes (e.g. physical activity).

For peer review only - http://bmjopen.bmj.com/site/about/guidelines.xhtml

INTRODUCTION

Advancements in the clinical management of cervical cancer have resulted in improved survival,[1,2] leading to a change in focus to treatment-related sequelae and survivorship issues, to reduce iatrogenic morbidity and to improve patient's long-term health.[3] Although many patients diagnosed with a cervical cancer have early stage disease (stage IA), which can be successfully treated with local excision, 64% of women having at least stage IB disease at diagnosis.[4] As a result, many patients will need to undergo radical surgery and/or chemoradiotherapy, that consequentially leads to the loss of fertility and an early menopause.[3]

The long-term effects of radiotherapy on the vagina,[5] bladder,[6] and bowel can occur in up to 30 to 40% of patients and can result in distressing symptoms, urinary/fecal incontinence [7] and tenesmus [8]. It can also lead to permanent physical changes, vaginal shortening/stenosis and reduced clitoral sensitivity, thereby physically limiting the ability to have and enjoy intercourse.[9] Chronic fatigue and pelvic insufficiency fractures are also frequently reported following radiotherapy treatment for cervical cancer, the symptoms of which may not diminish with time,[10] and can have a negative impact on daily functions and social quality of life.[11]

There is also substantial evidence of the devastating psychological consequences of a cervical cancer diagnosis. A high proportion of patients experience long-term psychological distress following their diagnosis,[12] which can impact on their social, work and functional well-being. The psychological impact is more pronounced in younger patients and those who receive chemoradiotherapy,[13] with reported lower levels of self- confidence and a more negative body image compared to those who undergo surgery alone.[14] Hence, a greater focus

For peer review only

on cervical cancer survivorship is warranted to support patients who are living alongside the physical, psychological and social impacts of cervical cancer treatments.

Identifying and implementing ways to manage treatment side-effects can be challenging, given the individualistic and complex nature of the survivorship experience,[15]. Moreover, concerns have been raised regarding the knowledge provision of treatment-related morbidity and clinical support services.[16] Physical activity (PA) has been shown to be a safe avenue to improve quality of life following cancer treatment,[17] with qualitative data indicating that PA can specifically improve physical, psychological, social and spiritual domains.[18] However, PA levels are reportedly low and remain low up to three years following treatment for a gynaecological malignancy.[19] In particular, cervical cancer survivors meet recommended PA guidelines less frequently than patients following other cancer types.[20] Additionally, exercise recommendations for cervical cancer survivors do not exist, thus supporting the need for tailored PA promotion in this population.

To date, there has been little research focus on promoting PA amongst those treated for cervical cancer. This is highlighted by cervical cancer cases being underrepresented in meta-analysis studies of gynaecological cancer.[17] Literature based in the broader context of gynaecological cancer and PA does provide some degree of insight regarding PA preferences and feasibility of different types of PA. Such studies suggest that PA which: incorporates home-based walking,[21–23] and gym activities;[23] is graded and flexible;[22] is combined with a form of support or counselling;[22] includes social interaction with peers,[24] tends to be favourable among survivors of gynaecological cancers. There is growing support for technology-based PA promotion among cancer survivors,[25] which can place less burden on participants, and can be easily tailored to the individual. However, the PA barriers faced after cervical cancer may differ to other gynaecological cancers due to variations in patient characteristics (e.g age)

For peer review only - http://bmjopen.bmj.com/site/about/guidelines.xhtml

and treatment side effects. Therefore, it is important to consider the specific needs of cervical cancer survivors during intervention design and implementation.

To the best of our knowledge, an intervention which promotes PA participation following treatment for cervical cancer has not been evaluated. Therefore, we aim to test the feasibility and acceptability of conducting and evaluating a multi-component intervention programme, informed by relevant theories of behaviour change, to increase PA levels following treatment for cervical cancer. This intervention has named ACCEPTANCE (Acceptability in Cervical Cancer of an Exercise-based Programme delivered Through An online Community Environment). This intervention was developed using the intervention mapping protocol.[26]

Aim

The overall aim of this study is to assess the feasibility and acceptability of a PA intervention following treatment for cervical cancer.

Objectives

1. To determine participant recruitment, retention and attrition rates
2. To review descriptive data to determine the feasibility of the inclusion criteria
3. To determine compliance and completeness of evaluation measures at all time points
4. To report the feasibility of delivering the intervention and evaluation measures
5. To explore participant compliance with the intervention
6. To obtain views on the acceptability of the intervention and evaluation measures
7. To describe device- assessed PA levels and questionnaire data outcomes of interest

For peer review only - http://bmjopen.bmj.com/site/about/guidelines.xhtml

METHODS AND ANALYSIS

Study design

This is a single- arm pre and post study to assess the feasibility and acceptability of conducting and evaluating a PA intervention following treatment for cervical cancer. A process evaluation will also be conducted throughout the study.

Study setting

This study will be conducted through the University Hospitals of Leicester (UHL) NHS Trust, in collaboration with Loughborough University and the University of Leicester.

Patient and Public Involvement (PPI)

PPI consisted of two groups of women treated for cervical cancer within the previous 10 years. PPI input has informed the development of the study since its inception. Insights and feedback gathered from one to one and group discussions with PPI members enabled development of the research question, assessment of the potential burden on participants and informed the intervention content to ensure that intervention materials were user friendly (Table 1)[27]. Continuation of PPI involvement will inform the intervention delivery and dissemination of results to the wider community as appropriate.

For peer review only - http://bmjopen.bmj.com/site/about/guidelines.xhtml

Table 1. PPI involvement and engagement in research activities

Research stage	Involvement	Mode
Research conception and design	Establishment of unmet needs after cervical cancer	In person group discussion PPI+RT
	Sharing personal experience of recovery after cervical cancer	One to one conversation PPI+SC
Recruitment for feasibility trial	PPI members provide feedback on recruitment poster	Email correspondence PPI+SC
	PPI members share study details via word of mouth or poster on online survivorship group	Email correspondence PPI+SC
Intervention design	PPI group to provide feedback on the feasibility of the intervention timeline	Group discussion between PPI + MR; use of questionnaire to elicit feedback
	PPI members to complete evaluation questionnaire and provide feedback on length and language use	One to one in person conversation PPI+SC
	PPI group to evaluate Intervention materials (diary, education and *Fitbit* guide)	Email correspondence PPI+SC
	PPI group to complete intervention launch session and provide feedback	Virtual group discussion PPI+SC
Programme delivery	PPI group to feedback on progress of intervention at mid-intervention delivery	Virtual group discussion PPI+SC
Dissemination of results	PPI group to feedback on appropriate strategies to disseminate results to the wider public (inc. patients and survivors)	Virtual group discussion PPI+SC

KEY: PPI= Patient and Public Involvement RT= Research Team; SC=Study Co-ordinator; TBC= to be confirmed

For peer review only

For peer review only - http://bmjopen.bmj.com/site/about/guidelines.xhtml

Participants and recruitment

Participants between the ages of 18 and 60 years who have undergone treatment for cervical cancer at least 6 months previously with curative intent (either surgery, chemoradiotherapy or both modalities), who do not meet the national PA guidelines (at least 150 minutes of moderate to vigorous intensity PA per week) [28], who can gain permission from their gynaecologist/ oncologist to take part and are fluent in English will be recruited. Exclusion criteria are benign or pre-malignant (cervical interepithelial neoplasia) disease of the cervix; clinical/radiological evidence of disseminated malignancy; pregnancy or breastfeeding; performance status ≥3; co-morbidity that in the opinion of the patient's supervising oncologist that would preclude the patient from meeting the study requirements.

Initial data base screening will take place by the clinical gynaecological oncology team to identify candidates who are at an appropriate age and stage in their treatment journey. Potentially eligible participants will be approached through their clinical team, recruitment posters will be placed in the gynaecological and oncology outpatient departments at UHL and study details (overview of research, inclusion criteria and relevant contact details) will be posted on social media platforms. Local cervical cancer support groups will also be contacted and given information on the study to raise awareness of the research. Screening will take place via a telephone call with a member of the research team. The Scottish Physical Activity Questionnaire [29] will be used to determine PA levels. Participants will be asked how many minutes per week they spend being active at a moderate intensity in the context of the last 7 days. Consent procedures will take place either virtually via video/ telephone call or in person at UHL.

For peer review only

For peer review only - http://bmjopen.bmj.com/site/about/guidelines.xhtml

Sample Size

This study will aim to recruit approximately 30 participants. In line with recommendations from the National Institute for Health Research (NIHR), a sample size of 30 is appropriate to answer the questions posed by a feasibility trial.[30] The feasibility of lifestyle and PA interventions in endometrial and breast cancer survivors have successfully employed similar sample sizes.[31,32]

Intervention

The aim of the multi-component intervention is to increase PA levels of the target population, specifically, individual and group-based walking over 12 consecutive weeks (Figure 1). Table 2 provides a description of each intervention component whilst Figure 2 outlines the Logic Model. Walking has been identified as a common PA preference among gynaecological cancer survivors[33], particularly for those who do not meet the recommended PA guidelines.[34] The intervention components are underpinned by social cognitive theory (SCT),[35] and are informed by the health belief model,[36] and theories of self-regulation.[26] Self-monitoring behaviour is strongly linked with successful PA behaviour change, particularly when combined with at least one other self-regulatory component.[37] The intervention includes education provision, problem solving and goal setting in relation to increasing PA levels after treatment for cervical cancer. Self-monitoring of PA behaviour will be facilitated by providing participants with an intervention diary to complete daily and weekly, fortnightly online health coaching via video or telephone call and a consumer PA self-monitoring device to receive real time feedback on their activity levels and prompts to be more active and to review goals. Throughout, peer support and group walking amongst participants will be encouraged via a messaging platform which will allow participants to maintain contact and organise group walking sessions. The study co-ordinator will suggest appropriate walk locations convenient to each group.

For peer review only - http://bmjopen.bmj.com/site/about/guidelines.xhtml

Table 2. Description of intervention components

Intervention component	Description
Intervention launch	**Education** (interactive session facilitated by the study co-ordinator, delivered virtually)<br>- What is meant by PA?<br>- What kinds of activities count as PA?<br>- What does moderate intensity PA feel like?<br>- What benefits are associated with PA?<br>- Knowledge of PA recommendations<br>- How many steps per day should you aim for?<br>**Barrier Identification**<br>- Is there anything that might stop you from being physically active?<br>- How can you overcome these challenges?<br>**Goal Setting**<br>- Introduction to SMART goals<br>- Using *Fitbit inspire* to set goals related to steps and intensity<br>*Each section divided by guided 'stand up and stretch' prompts
Self-monitoring of PA	- The PA monitor will provide participants with feedback on their activity levels via: number of steps taken by participants and the number of minutes that participants spend in a moderate intensity activity each day.
Prompts	- Prompts offered by PA monitor to encourage PA<br>- Prompts to update participant on their progress in reaching their daily goal.
Intervention diary (monitoring of well-being)	**Daily entries**<br>- Input daily step count as shown on Fitbit<br>- Rate mood out of 10<br>**Weekly entries**<br>- Rate the following symptoms on a traffic light system: energy levels, anxiety levels, body confidence, physical pain, bladder issues.
Health coaching	- Revision of previous goal(s) and the outcome (successful/ unsuccessful); attribute reasons for this outcome<br>- Identification of challenges and facilitators regarding previous goal<br>- Revision of steps, intensity and well-being over the previous two weeks<br>- Facilitated goal setting and identification of potential challenges for the following 2 weeks
Group messaging	- To be used as a platform to schedule group walks (organising a time and a place) with the aim of one group walk per week. Standardised communication prompts to be offered by the study co-ordinator.
Online community	- Participants will be encouraged to utilise the study specific community on the PA monitor application, accessed via smart phone where personal PA insights and statistics can be shared with other participants.

1

2

For peer review only - http://bmjopen.bmj.com/site/about/guidelines.xhtml

Measures

All PA and health measures will be assessed at baseline, week-12 and 3- months after completion (week-24). Previous literature suggests that psychological measures may take longer to change than physical measures (e.g., menopausal symptoms or PA) and therefore to reduce participant burden, only these will be taken at week- 6 (table 3).

Table 3. PA and health outcomes, outcome measures and measurement time points

**Measures**	**Assessment tool**	**Baseline**	**Week 6**	**Week 12**	**Week 24**
Device- assessed PA	Accelerometer	x	x	x	x
Menopausal Symptoms	MRS	x	x	x	x
Quality of life	EORTC-QLQ-30	x		x	x
Depression and Anxiety	HADS	x		x	x
Fatigue	MSFI-SF	x		x	x
Walking Self- efficacy	Self- efficacy for Walking scale	x		x	x
Motivation for Exercise	BREQ-3	x		x	x
Enjoyment of PA	PACES	x		x	x

PA and health measures

An accelerometer (GENEActiv, Activinsights Ltd) and sleep log will be administered at all evaluation time points to measure PA. Participants will be asked to wear the accelerometer on their non-dominant wrist 24/7 for 8 days at each time point. Valid data will be at least 3 days >16 hours) of accelerometer data collected.(e.g. [38]) Device assessed PA will the primary research outcome measure of interest in the subsequent pilot study and definitive main trial.

Participants' belief in their ability to successfully complete incremental 5 to 50- minute periods of walking at a moderate/ brisk pace will be assessed via the self-efficacy for walking scale [39]. Motivation for PA will be assessed using the behavioural regulation in exercise

For peer review only - http://bmjopen.bmj.com/site/about/guidelines.xhtml

questionnaire (BREQ-3).[40] This is a 23- item scale which assesses motivation on a self-determination continuum. Enjoyment of PA will also be assessed via the original 18- item PACES scale.[41]

In relation to well- being, menopausal symptoms, such as hot flushes and sweating will be investigated using the menopausal rating Scale (MRS).[42] Quality of life will be assessed using the EORTC QLQ- 30, which is commonly used in gynaecological cancer populations. It includes 5 functional scales, 3 symptom scales, a global health scale and a quality of life scale. The Hospital Anxiety and Depression scale (HADS),[43] will be administered to measure symptoms of anxiety and depression. Fatigue will be assessed using the Multi- dimensional fatigue symptom inventory (MFSI-SF),[44] a measure which has been previously used to assess fatigue in a gynaecological cancer population.[21]

Evaluation related feasibility measures

Participant recruitment rates will be monitored by recording the number of identified potential participants, the number of those who express an interest in taking part, the number of those who are eligible and not eligible along with the reasons for ineligibility and the number who consent to participate. Retention and attrition rates will be monitored by recording the number of participants who withdraw from the study and the number who do not attend follow-up. Where possible, reasons for withdrawal will also be reported. The number of participants who complete the PA and health measures at each time point will be summarised.

Process evaluation

The process evaluation will be conducted to explore participant experiences of the study and intervention as well as compliance with the intervention components, to identify facilitators and barriers to increasing PA and any suggestions for improvement and refinement. Compliance with intervention components will be reviewed throughout the intervention. Data

For peer review only - http://bmjopen.bmj.com/site/about/guidelines.xhtml

will be presented on launch attendance rates, the number of diary entries completed, number of daily step counts recorded to reflect compliance of wearing the PA monitor, number of health coaching sessions attended, the number of participants who post on the online forum and engage in group messaging, the number of participants who take part in group walks and the frequency of these.

Questionnaires will be administered to understand participant's experience of attending the intervention launch, of using the PA monitor and the group messaging. A semi- structured interview will also be conducted by the study co-ordinator to further explore perceptions of the intervention and the evaluation measures, perceived benefits of participation, maintenance of the intervention components and PA levels. Interview data will be analysed using Template Analysis,[45] employing a deductive coding framework to inform study refinement. To ensure a balanced insight and that subsequent meaningful refinement can be implemented, some transcripts will be coded by an independent researcher who did not contribute to the intervention design.

A researcher log will be completed by the study co-ordinator where a record of interactions with participants (e.g. email conversations/ telephone conversations) will be kept throughout the duration of the study. Feedback will be broadly divided into four categories: evaluation of intervention components; experience of evaluation measures; alterations to future intervention implementation; barriers to PA and reasons for discontinuation (where relevant).

Data collection

The accelerometer and the quantitative measures will be posted to participants. Process evaluation questionnaires will be completed online throughout the intervention. The evaluation interview will be offered after the week-24 time point to all participants (including those who have withdrawn) either in person or via video call.

For peer review only - http://bmjopen.bmj.com/site/about/guidelines.xhtml

Statistical Analysis

As this is a feasibility study, main analyses will utilise descriptive statistics. Data will be analysed using *IBM SPSS statistics 25*.

The feasibility of inclusion criteria

We have suggested that if an eligibility criterion accounts for 40% of ineligibility, it will be reported.

The feasibility of study recruitment and retention

A priori, we have defined a recruitment success rate as recruitment of 30 participants in the first 3- months or that 75% of those identified as eligible are recruited, and a successful retention rate at week-24 as 70%.

The feasibility of and compliance with intervention components

Attendance rates at the intervention launch, engagement with the online forum, frequency of wearing the PA monitor, frequency of group walking sessions, compliance with diary completion and attendance rates at health coaching sessions will all be summarised. The threshold of feasibility for these rates will be 70% of participants complying respectively.

The feasibility of the evaluation measures

Compliance with evaluation measures will be deemed successful if no more than 20% of participants fail to provide questionnaire and accelerometer data at both baseline and week-24 follow-up.

Physical activity data and questionnaire outcomes

Data from the accelerometer will be cleaned and processed in GGIR using *R programming* and analysed using *IBM SPSS statistics 25*. Means and standard deviation (SD) will be used to describe the PA from the accelerometer at each evaluation time point. Constructs measured

For peer review only - http://bmjopen.bmj.com/site/about/guidelines.xhtml

via questionnaire will be analysed by standard scoring procedures. Questionnaire outcomes of interest will be reported on means (or medians where necessary) and SDs.

Ethics and dissemination

Ethical approval has been granted by the West of Scotland Research Ethics Committee 1 for this study. Results will primarily be used to refine intervention components and to inform a larger full-scale trial, if appropriate. Results from the study will be shared in peer reviewed journals, presented at international conferences and will be shared with the University Hospitals of Leicester and Leicester Hospital's charity.

For peer review only

DISCUSSION

This article describes the protocol for a study testing the feasibility and acceptability of conducting and evaluating of a multi- component PA intervention following treatment for cervical cancer. Regular PA has been shown to have physical and psychological benefits and to be safe after cancer .[46] PA can also alleviate some of the symptoms experienced as a result of cancer treatment .[21] PA levels appear to be low following cervical cancer [20] and this may be partly due to the lack of a specific intervention that takes into consideration the particular side-effects that can occur, in particular pelvic pain and incontinence, that may not be such an issue in other cancer types.

The study protocol details a 12-week intervention programme to promote walking and PA, with evaluation measures taken at baseline, 6- weeks, 12 weeks and 24 weeks. The process evaluation is a mix of questionnaires and a qualitative interview. The intervention was designed using the intervention mapping protocol.[26] Development followed a systematic 6-step framework which prioritises input from the target population as well as integrating behaviour change theories (BCTs) and constructs to underpin change within the intervention, informed by relevant literature.[47] Interventions underpinned by relevant BCTs have been found to be more successful in implementing change and in identifying more specific evaluation criteria.[46]

The current PA guidelines offered by the Government Chief Medical Officers do not specify recommendations for cervical cancer survivors.[28] However, using a development framework that prioritises insights and experiences from the target population is a great strength of this intervention, as it allowed direct and effective tailoring of the intervention components despite specific recommendations not being available. Hence, the aerobic PA recommendations were deemed an appropriate target for women treated for cervical cancer.[27] Additionally, the criterion to include women who are at least 6- months post treatment was also a decision

For peer review only - http://bmjopen.bmj.com/site/about/guidelines.xhtml

informed by the need's assessment, with the time between 3 and 9 months post treatment being identified as critical for intervention and for positive change.[48] A limitation is that the protocol does not incorporate strength training, and therefore does not reflect the current PA guidelines for after cancer. However, as PA levels are generally low following cervical cancer treatment, it seemed appropriate to promote one PA modality which was realistically achievable (i.e. walking) to enhance adherence to the intervention.

This study will add to the current debate regarding the role of technology in promoting PA after cancer. The intervention utilises a PA monitor and its associated online community and a messaging platform. Previous research supports the use of such e-health technologies in combination with health coaching over a shorter duration (4-week programme).[49] However, it has been suggested that further investigation is needed to test the acceptability of such technologies in more diverse populations,[50] to explore optimal intervention length and to question the maintenance of PA behaviour change.[49] Although a limitation of this protocol is that statistically relevant changes in PA between time points will not be reported, trends in PA levels will provide data inferring the feasibility of promoting and maintaining PA behaviours. The process evaluation will also provide valuable insight regarding the acceptability of the components.

For peer review only

For peer review only - http://bmjopen.bmj.com/site/about/guidelines.xhtml

CONCLUSION

We described the protocol of a study which tests the feasibility and acceptability of delivering and evaluating a multi- component physical activity intervention for women following treatment for cervical cancer. A process evaluation plan will provide insight into whether the intervention components and evaluation measures are accepted by participants. Findings from the study will inform intervention refinement in preparation for a full-scale pilot trial.

For peer review only

For peer review only - http://bmjopen.bmj.com/site/about/guidelines.xhtml

Contributorship statement

All authors contributed to the study conception and design and protocol preparation. The first draft of the manuscript was written by NM and EM. CE, FM and HJM significantly revised the manuscript. All authors read and approved the final manuscript.

Competing Interest statement

ELM has received research grants from Intuitive Surgical and Hope Against Cancer for unrelated work. CLE has received lecture fees for GSK and is on the clinical advisory board for Inivata.

Funding Statement

This research is supported by the National Institute for Health Research (NIHR) Leicester Biomedical Research Centre, which is a partnership between University Hospitals of Leicester NHS Trust, Loughborough University and the University of Leicester, UK. Support. Funding was provided by the Economic, Social and Research Council (ES/P000711/1), Loughborough university (ES/P000711/1), and the Leicester Hospital's charity, University Hospitals of Leicester (Q843).

Acknowledgements

The authors are very grateful to the members of the Patient and Public Involvement group for their contributions and insights throughout the intervention development stages.

For peer review only - http://bmjopen.bmj.com/site/about/guidelines.xhtml

REFERENCES

1 CRUK U. Cervical cancer statistics. 2017.

2 Vale CL, Tierney JF, Davidson SE, *et al.* Substantial Improvement in UK Cervical Cancer Survival with Chemoradiotherapy: Results of a Royal College of Radiologists' Audit. *Clin Oncol* Published Online First: 2010. doi:10.1016/j.clon.2010.06.002

3 Moss EL, Taneja S, Munir F, *et al.* Iatrogenic Menopause After Treatment for Cervical Cancer. *Clin Oncol* 2016;**28**:766–75. doi:10.1016/j.clon.2016.08.016

4 Public Health England. Cervical cancer screening: invasive cervical cancer audit 2013 to 2016. 2019.https://www.gov.uk/government/publications/cervical-screening-invasive-cervical-cancer-audit-2013-to-2016/executive-summary#staging-information

5 Bergmark K, Åvall-Lundqvist E, Dickman PW, *et al.* Patient-rating of distressful symptoms after treatment for early cervical cancer. *Acta Obstet Gynecol Scand* Published Online First: 2002. doi:10.1034/j.1600-0412.2002.810512.x

6 Pisarska M, Sajdak S. Lower urinary tract function after postoperative radiotherapy in the treatment of cervical cancer. *Eur J Gynaecol Oncol* 2003.

7 Sood AK, Nygaard I, Shahin MS, *et al.* Anorectal dysfunction after surgical treatment for cervical cancer. *J Am Coll Surg* Published Online First: 2002. doi:10.1016/S1072-7515(02)01311-X

8 Butler-Manuel SA, Summerville K, Ford A, *et al.* Self-assessment of morbidity following radical hysterectomy for cervical cancer. *J Obstet Gynaecol (Lahore)* Published Online First: 1999. doi:10.1080/01443619965552

9 Jensen PT, Groenvold M, Klee MC, *et al.* Longitudinal study of sexual function and vaginal changes after radiotherapy for cervical cancer. *Int J Radiat Oncol Biol Phys* Published Online First: 2003. doi:10.1016/S0360-3016(03)00362-6

10 Elghamrawi KA, Haggag MH, Habib EE. Treatment complications among long-term survivors of cervical cancer: Treated by surgery or radiotherapy. Oncol. Rev. 2007. doi:10.1007/s12156-011-0093-0

11 Schover LR, Fife M, Gershenson DM. Sexual dysfunction and treatment for early stage cervical cancer. *Cancer* Published Online First: 1989. doi:10.1002/1097-0142(19890101)63:1<204::AID-CNCR2820630133>3.0.CO;2-U

12 Le Borgne G, Mercier M, Woronoff AS, *et al.* Quality of life in long-term cervical cancer survivors: A population-based study. *Gynecol Oncol* 2013;**129**:222–8. doi:10.1016/j.ygyno.2012.12.033

13 Derks M, Van Lonkhuijzen LRCW, Bakker RM, *et al.* Long-Term Morbidity and Quality of Life in Cervical Cancer Survivors: A Multicenter Comparison between Surgery and Radiotherapy as Primary Treatment. *Int J Gynecol Cancer* Published Online First: 2017. doi:10.1097/IGC.0000000000000880

14 Kirchhoff AC, Yi J, Wright J, *et al.* Marriage and divorce among young adult cancer survivors. *J Cancer Surviv* Published Online First: 2012. doi:10.1007/s11764-012-0238-6

For peer review only - http://bmjopen.bmj.com/site/about/guidelines.xhtml

15 Sekse RJT, Raaheim M, Blaaka G, *et al.* Life beyond cancer: Women's experiences 5-years after treatment for gynaecological cancer. *Scand J Caring Sci* 2010;**24**:799–807. doi:10.1111/j.1471-6712.2010.00778.x

16 Faller H, Brähler E, Härter M, *et al.* Unmet needs for information and psychosocial support in relation to quality of life and emotional distress: A comparison between gynecological and breast cancer patients. *Patient Educ Couns* 2017;**100**:1934–42. doi:10.1016/j.pec.2017.05.031

17 Buffart LM, Kalter J, Sweegers MG, *et al.* Effects and moderators of exercise on quality of life and physical function in patients with cancer: An individual patient data meta-analysis of 34 RCTs. *Cancer Treat Rev* 2017;**52**:91–104. doi:10.1016/j.ctrv.2016.11.010

18 Burke S, Wurz A, Bradshaw A, *et al.* Physical activity and quality of life in cancer survivors: A meta-synthesis of qualitative research. Cancers (Basel). 2017. doi:10.3390/cancers9050053

19 Fleming S, Jones T, Janda M, *et al.* Physical activity trajectories following gynecological cancer: results from a prospective, longitudinal cohort study. *Int J Gynecol Cancer* Published Online First: 2020. doi:10.1136/ijgc-2020-001543

20 Thraen-Borowski KM, Gennuso KP, Cadmus-Bertram L. Accelerometer-derived physical activity and sedentary time by cancer type in the United States. *PLoS One* Published Online First: 2017. doi:10.1371/journal.pone.0182554

21 Donnelly CM, Blaney JM, Lowe-Strong A, *et al.* A randomised controlled trial testing the feasibility and efficacy of a physical activity behavioural change intervention in managing fatigue with gynaecological cancer survivors. *Gynecol Oncol* 2011;**122**:618–24. doi:10.1016/j.ygyno.2011.05.029

22 Donnelly CM, Lowe-Strong A, Rankin JP, *et al.* A focus group study exploring gynecological cancer survivors' experiences and perceptions of participating in a RCT testing the efficacy of a home-based physical activity intervention. *Support Care Cancer* 2013;**21**:1697–170. doi:10.1007/s00520-012-1716-0

23 Brown JC, John GM, Segal S, *et al.* Physical activity and lower limb lymphedema among uterine cancer survivors. *Med Sci Sports Exerc* 2013;**45**:2091. doi:10.1249/MSS.0b013e318299afd4

24 Rossi A, Garber CE, Ortiz M, *et al.* Feasibility of a physical activity intervention for obese, socioculturally diverse endometrial cancer survivors. *Gynecol Oncol* 2016;**142**:304–10. doi:10.1016/j.ygyno.2016.05.034

25 Rossi A, Frechette L, Miller D, *et al.* Acceptability and feasibility of a Fitbit physical activity monitor for endometrial cancer survivors. *Gynecol Oncol* 2018;**149**:470–5. doi:10.1016/j.ygyno.2018.04.560

26 Bartholomew, Eldredge L Highfield L, Hartman M, Mullen P, *et al.* Using Intervention Mapping to Adapt Evidence-Based Interventions. In: *Planning health promotion programs : an intervention mapping approach*. 2016.

27 Millet N, McDermott HJ, Moss EL, *et al.* Increasing physical activity levels following treatment for cervical cancer: an intervention mapping approach. *J Cancer Surviv* Published Online First: 2021. doi:10.1007/s11764-021-01058-y

For peer review only - http://bmjopen.bmj.com/site/about/guidelines.xhtml

28 Davies DSC, Atherton F, McBride M, *et al.* UK Chief Medical Officers' Physical Activity Guidelines. 2019.

29 Lowther M, Mutrie N, Loughlan C, *et al.* Development of a Scottish physical activity questionnaire: A tool for use in physical activity interventions. *Br J Sports Med* Published Online First: 1999. doi:10.1136/bjsm.33.4.244

30 Lancaster GA, Dodd S, Williamson PR. Design and analysis of pilot studies: Recommendations for good practice. J. Eval. Clin. Pract. 2004. doi:10.1111/j..2002.384.doc.x

31 Rossi A, Frechette L, Miller D, *et al.* Acceptability and feasibility of a Fitbit physical activity monitor for endometrial cancer survivors. *Gynecol Oncol* 2018;**149**:470–5. doi:10.1016/j.ygyno.2018.04.560

32 Pullen T, Sharp P, Bottorff JL, *et al.* Acceptability and satisfaction of project MOVE: A pragmatic feasibility trial aimed at increasing physical activity in female breast cancer survivors. *Psychooncology* Published Online First: 2018. doi:10.1002/pon.4662

33 Farrokhzadi L, Dhillon HM, Goumas C, *et al.* Physical Activity Correlates, Barriers, and Preferences for Women with Gynecological Cancer. In: *International Journal of Gynecological Cancer*. 2016. doi:10.1097/IGC.0000000000000790

34 Stevinson C, Tonkin K, Capstick V, *et al.* A Population-Based Study of the Determinants of Physical Activity in Ovarian Cancer Survivors. *J Phys Act Heal* 2009;**6**:339–46. doi:10.1123/jpah.6.3.339

35 Bandura A. *Social foundations of thought and action : a social cognitive theory / Albert Bandura.* 1986.

36 Janz K, Becker MH. The Health Belief Model : A Decade Later. 1984;:1–47.

37 Michie S, Abraham C, Whittington C, *et al.* Effective Techniques in Healthy Eating and Physical Activity Interventions: A Meta-Regression. *Heal Psychol* Published Online First: 2009. doi:10.1037/a0016136

38 Yates T, Griffin S, Bodicoat DH, *et al.* PRomotion Of Physical activity through structured Education with differing Levels of ongoing Support for people at high risk of type 2 diabetes (PROPELS): Study protocol for a randomized controlled trial. *Trials* Published Online First: 2015. doi:10.1186/s13063-015-0813-z

39 McAuley E, Blissmer B, Katula J, *et al.* Exercise environment, self-efficacy, and affective responses to acute exercise in older adults. *Psychol Heal* Published Online First: 2000. doi:10.1080/08870440008401997

40 Markland D, Tobin V. A modification to the behavioural regulation in exercise questionnaire to include an assessment of amotivation. *J Sport Exerc Psychol* Published Online First: 2004. doi:10.1123/jsep.26.2.191

41 Kendzierski D, DeCarlo KJ. Physical Activity Enjoyment Scale: Two Validation Studies. *J Sport Exerc Psychol* Published Online First: 2016. doi:10.1123/jsep.13.1.50

42 Hauser GA, Huber IC, Keller PJ, *et al.* EVALUATION DER KLIMAKTERISCHEN BESCHWERDEN (MENOPAUSE RATING SCALE [MRS]). Zentralbl. Gynakol. 1994.

For peer review only - http://bmjopen.bmj.com/site/about/guidelines.xhtml

43 Zigmond, Anthony S, Snaith, Philip R. The Hospital Anxiety and Depression Scale. *Acta Psychiatr Scand* 1983;**67**:361–70.

44 Stein KD, Jacobsen PB, Blanchard CM, *et al.* Further validation of the multidimensional fatigue symptom inventory-short form. *J Pain Symptom Manage* Published Online First: 2004. doi:10.1016/j.jpainsymman.2003.06.003

45 King N. Doing Template Analysis. In: *Qualitative Organizational Research: Core Methods and Current Challenges*. 2017. doi:10.4135/9781526435620.n24

46 Turner RR, Steed L, Quirk H, *et al.* Interventions for promoting habitual exercise in people living with and beyond cancer. *Cochrane Database Syst Rev* Published Online First: 2018. doi:10.1002/14651858.CD010192.pub3

47 Hirschey R, Bryant AL, Macek C, *et al.* Predicting physical activity among cancer survivors: Meta-analytic path modeling of longitudinal studies. *Heal Psychol* Published Online First: 2020. doi:10.1037/hea0000845

48 Sabiston CM, Brunet J, Vallance JK, *et al.* Prospective Examination of Objectively Assessed Physical Activity and Sedentary Time after Breast Cancer Treatment : Sitting on the Crest of the Teachable Moment. 2014;**23**:1324–31. doi:10.1158/1055-9965.EPI-13-1179

49 Gell NM, Grover KW, Humble M, *et al.* Efficacy, feasibility, and acceptability of a novel technology-based intervention to support physical activity in cancer survivors. *Support Care Cancer* Published Online First: 2017. doi:10.1007/s00520-016-3523-5

50 Haberlin C, O'Dwyer T, Mockler D, *et al.* The use of eHealth to promote physical activity in cancer survivors: a systematic review. Support. Care Cancer. 2018. doi:10.1007/s00520-018-4305-z

For peer review only

Protocol amendments

Any modifications to the protocol will be approved by the West of Scotland Research Ethics Committee 1 prior to implementation and notified to the health authorities in accordance with local regulations.

Ethical approvals

Ethical approval was given by the West of Scotland Research Ethics Committee (20/WS/0062).

List of Figures

Figure 1. Intervention Programme Flowchart

*Figure 2***.** Logic Model for the ACCEPTANCE trial

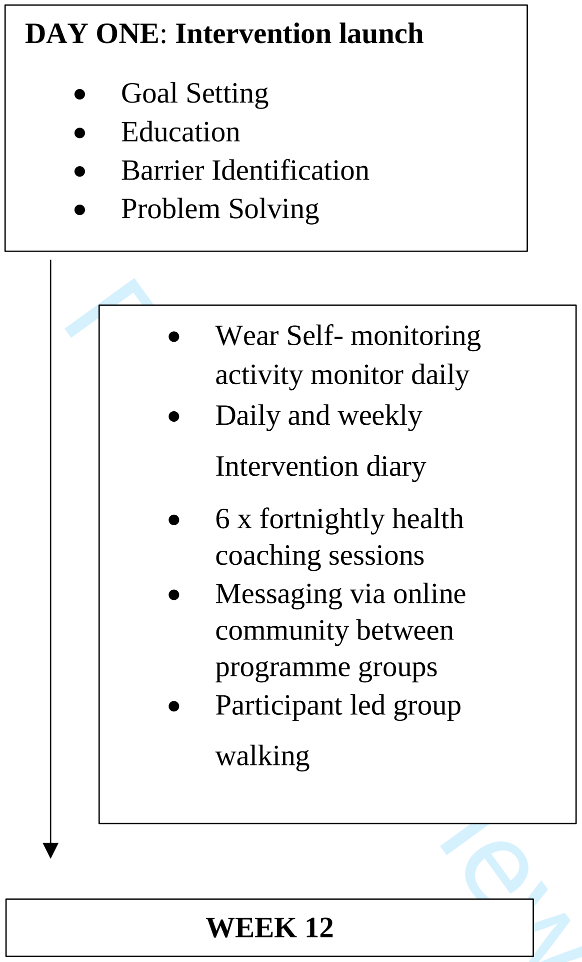

Figure 1. Intervention programme flowchart

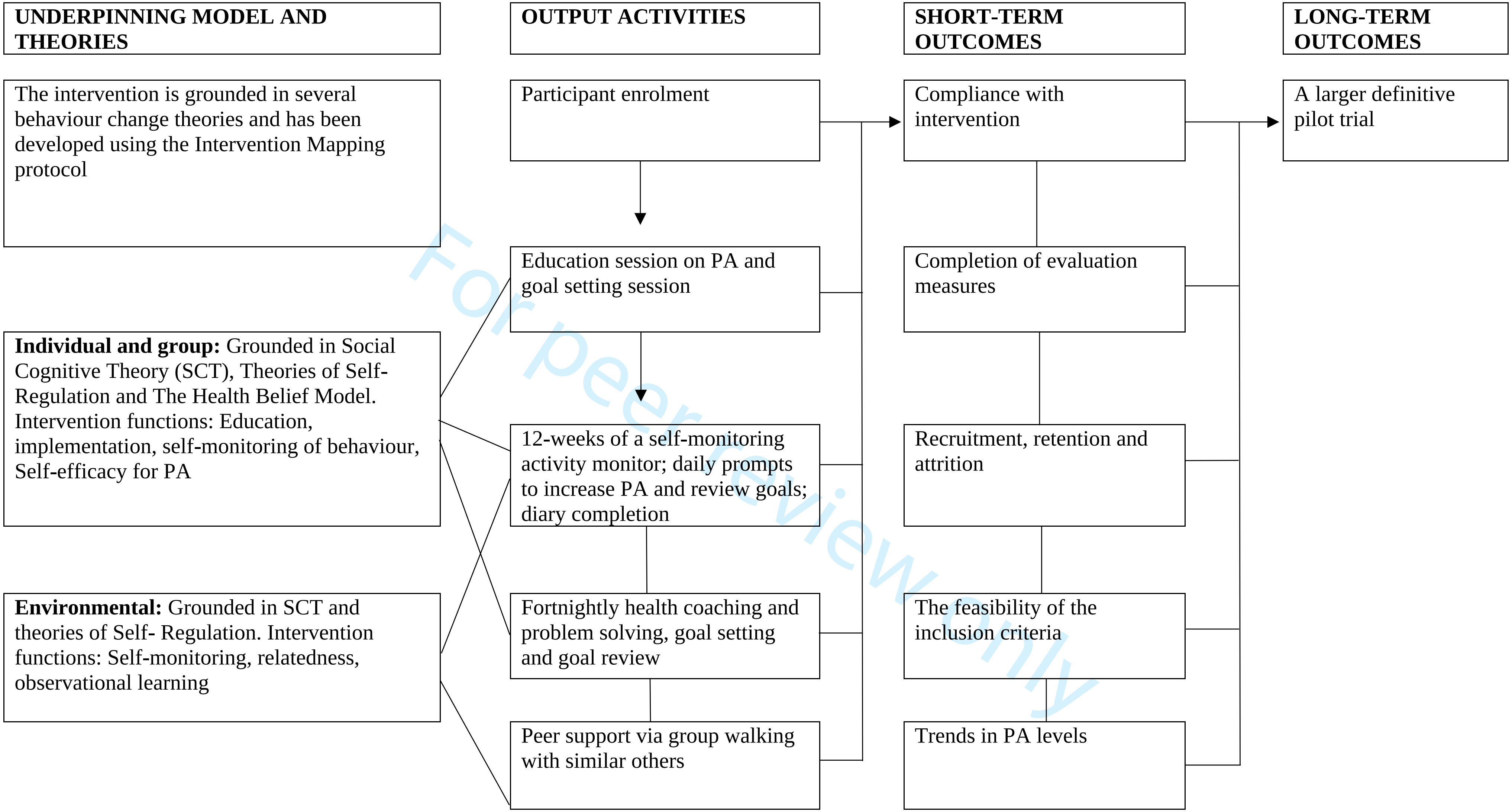

Figure 2. The logic model for the ACCEPTANCE study

For peer review only

BMJ Open

ACCEPTANCE: Protocol for a feasibility study of a multicomponent physical activity intervention following treatment for cervical cancer

Journal:	*BMJ Open*
Manuscript ID	bmjopen-2020-048203.R2
Article Type:	Protocol
Date Submitted by the Author:	10-Nov-2021
Complete List of Authors:	Millet, Nessa; Loughborough University School of Sport Exercise and Health Sciences<br>McDermott, Hilary J; Loughborough University School of Sport Exercise and Health Sciences<br>Munir, Fehmidah; Loughborough University School of Sport Exercise and Health Sciences, School of Sport, Exercise and Health Sciences<br>Edwardson, Charlotte; University of Leicester Diabetes Research Centre; NIHR Leicester Biomedical Research Centre<br>Moss, Esther; Leicester Cancer Research Centre, Leicester Cancer Research Centre
**Primary Subject Heading**:	Sports and exercise medicine
Secondary Subject Heading:	Oncology, Public health
Keywords:	Gynaecological oncology < GYNAECOLOGY, PUBLIC HEALTH, SOCIAL MEDICINE

SCHOLARONE™
Manuscripts

TITLE PAGE

Title: ACCEPTANCE: Protocol for a feasibility study of a multicomponent physical activity intervention following treatment for cervical cancer

Corresponding author: Dr Esther L Moss

Postal address: Leicester Cancer Research Centre, University of Leicester, Leicester, UK, LE2 7LX. Email: em321@le.ac.uk. TEL: +44 116 2523170 Fax: +44 116 258 8210

Authors: Nessa Millet[1], Hilary J McDermott[1], Fehmidah Munir[1], Charlotte L Edwardson[2,3] Esther L Moss[4]

[1]School of Sport, Exercise and Health Sciences, National Centre for Sport and Exercise Medicine, Loughborough University, Loughborough, UK

[2]Diabetes Research Centre, University of Leicester, Leicester, UK

[3]NIHR Leicester Biomedical Research Centre, Leicester, UK

[4]Leicester Cancer Research Centre, University of Leicester, Leicester, UK

Word count: 3,128

Key words: Gynaecological Oncology, Physical Activity, Quality of Life

For peer review only - http://bmjopen.bmj.com/site/about/guidelines.xhtml

ABSTRACT

Introduction: Cervical cancer treatment can have life changing sequelae and be associated with poor short- and long-term quality of life. Physical activity (PA; i.e. bodily movement) is known to improve health outcomes and quality of life for cancer survivors, both physically and psychologically. To date, no interventions to increase PA following cervical cancer have been evaluated. This study aims to (1) determine the feasibility of conducting a PA intervention after cervical cancer and (2) to explore the acceptability of the programme and evaluation measures.

Methods and analysis: The design is a pre and post study. Thirty participants aged between 18- 60 years from the Midlands region, United Kingdom, who have completed primary treatment for cervical cancer at least 6- months previously and do not meet the national PA guidelines will be recruited. Identification of potential participants will take place through the University Hospitals of Leicester National Health Service (NHS) Trust. Participants will receive an intervention focused on increasing PA through the provision of education, action planning, goal setting, problem solving and self-monitoring of PA behaviour, particularly steps per day. Device assessed PA and questionnaires will be completed at baseline, week-6, week-12 and week-24. Feasibility will be assessed in terms of recruitment, retention, attrition, completion of measures and intervention compliance for which, specific feasibility criteria have been established. The process evaluation will explore the experiences and acceptability of the intervention components and evaluation measures.

Ethics and dissemination: Ethical approval has been granted by the West of Scotland Research Ethics Committee 1 for this study. Results will inform intervention refinement for the design of a definitive pilot trial. These results will be disseminated via peer-reviewed publications and international conferences whilst input from a patient and public involvement (PPI) group will inform effective ways to circulate results amongst the wider community.

Registration details: ISRCTN16349793, Registered 30/09/2020.

For peer review only - http://bmjopen.bmj.com/site/about/guidelines.xhtml

ARTICLE SUMMARY

- The integration of behaviour change theory and insight from the target population to inform the development of practical strategies ensures an appropriate and user-friendly intervention.
- A mixed methods process evaluation will provide a great depth of understanding relating to the experiences and acceptability of the intervention and will enhance subsequent refinement where necessary.
- As this is a feasibility study, the analysis will not report statistically powered differences in outcomes (e.g. physical activity).

For peer review only - http://bmjopen.bmj.com/site/about/guidelines.xhtml

INTRODUCTION

Advancements in the clinical management of cervical cancer have resulted in improved survival,[1,2] leading to a change in focus to treatment-related sequelae and survivorship issues, to reduce iatrogenic morbidity and to improve patient's long-term health.[3] Although many patients diagnosed with a cervical cancer have early stage disease (stage IA), which can be successfully treated with local excision, 64% of women having at least stage IB disease at diagnosis.[4] As a result, many patients will need to undergo radical surgery and/or chemoradiotherapy, that consequentially leads to the loss of fertility and an early menopause.[3]

The long-term effects of radiotherapy on the vagina,[5] bladder,[6] and bowel can occur in between 30 to 40% of patients and can result in distressing symptoms, urinary/fecal such as incontinence [7] and tenesmus [8]. It can also lead to permanent physical changes, vaginal shortening/stenosis and reduced clitoral sensitivity, thereby physically limiting the ability to have and enjoy intercourse.[9] Chronic fatigue and pelvic insufficiency fractures are also frequently reported following radiotherapy treatment for cervical cancer, the symptoms of which may not diminish with time,[10] and can have a negative impact on daily functions and social quality of life.[11]

There is also substantial evidence of the devastating psychological consequences of a cervical cancer diagnosis. A high proportion of patients experience long-term psychological distress following their diagnosis,[12] which can impact on their social, work and functional well-being. The psychological impact is more pronounced in younger patients and those who receive chemoradiotherapy,[13] with reported lower levels of self- confidence and a more negative body image compared to those who undergo surgery alone.[14] Hence, a greater focus

For peer review only - http://bmjopen.bmj.com/site/about/guidelines.xhtml

on cervical cancer survivorship is warranted to support patients who are living alongside the physical, psychological and social impacts of cervical cancer treatments.

Identifying and implementing ways to manage treatment side-effects can be challenging, given the individualistic and complex nature of the survivorship experience,[15]. Moreover, concerns have been raised regarding the knowledge provision of treatment-related morbidity and clinical support services.[16] Physical activity (PA), defined as *'any bodily movement produced by skeletal muscles that results in energy expenditure'* [17, p126] has been shown to be a safe avenue to improve quality of life following cancer treatment,[18] with qualitative data indicating that PA can specifically improve physical, psychological, social and spiritual domains.[19] However, PA levels are reportedly low and remain low up to three years following treatment for a gynaecological malignancy.[20] In particular, cervical cancer survivors meet recommended PA guidelines less frequently than patients following other cancer types.[21] Exercise recommendation for cancer survivors have been suggested [22], however these do not target the specific PA barriers faced after cervical cancer, thus supporting the need for tailored PA promotion in this population.

To date, there has been little research focus on promoting PA amongst those treated for cervical cancer. This is highlighted by cervical cancer cases being underrepresented in meta-analysis studies of gynaecological cancer.[18] Literature based in the broader context of gynaecological cancer and PA does provide some degree of insight regarding PA preferences and feasibility of different types of PA. Such studies suggest that PA which: incorporates home-based walking,[23–25] and gym activities;[25] is graded and flexible;[24] is combined with a form of support or counselling;[24] includes social interaction with peers,[26] tends to be favourable among survivors of gynaecological cancers. There is growing support for technology-based PA promotion among cancer survivors,[27] which can place less burden on participants, and

For peer review only - http://bmjopen.bmj.com/site/about/guidelines.xhtml

For peer review only

can be easily tailored to the individual. However, the PA barriers faced after cervical cancer may differ to other gynaecological cancers due to variations in patient characteristics (e.g age) and treatment side effects. Therefore, it is important to consider the specific needs of cervical cancer survivors during intervention design and implementation.

To the best of our knowledge, an intervention which promotes PA participation following treatment for cervical cancer has not been evaluated. Therefore, we aim to test the feasibility and acceptability of conducting and evaluating a multi-component intervention programme, informed by relevant theories of behaviour change, to increase PA levels following treatment for cervical cancer. This intervention has named ACCEPTANCE (Acceptability in Cervical Cancer of an Exercise-based Programme delivered Through An online Community Environment). This intervention was developed using the intervention mapping protocol.[28]

Aim

The overall aim of this study is to assess the feasibility and acceptability of a PA intervention following treatment for cervical cancer.

Objectives

1. To determine participant recruitment, retention and attrition rates
2. To review descriptive data to determine the feasibility of the inclusion criteria
3. To determine compliance and completeness of evaluation measures at all time points
4. To report the feasibility of delivering the intervention and evaluation measures
5. To explore participant compliance with the intervention
6. To obtain views on the acceptability of the intervention and evaluation measures
7. To describe device- assessed PA levels and questionnaire data outcomes of interest

For peer review only

For peer review only - http://bmjopen.bmj.com/site/about/guidelines.xhtml

METHODS AND ANALYSIS

Study design

This is a single- arm pre and post study to assess the feasibility and acceptability of conducting and evaluating a PA intervention following treatment for cervical cancer. A process evaluation will also be conducted throughout the study.

Study setting

This study will be conducted through the University Hospitals of Leicester (UHL) NHS Trust, in collaboration with Loughborough University and the University of Leicester.

Patient and Public Involvement (PPI)

PPI consisted of two groups of women treated for cervical cancer within the previous 10 years. PPI input has informed the development of the study since its inception. Insights and feedback gathered from one to one and group discussions with PPI members enabled development of the research question, assessment of the potential burden on participants and informed the intervention content to ensure that intervention materials were user friendly (Table 1)[29]. Continuation of PPI involvement will inform the intervention delivery and dissemination of results to the wider community as appropriate.

For peer review only

For peer review only - http://bmjopen.bmj.com/site/about/guidelines.xhtml

Table 1. PPI involvement and engagement in research activities

**Research stage**	**Involvement**	**Mode**
Research conception and design	Establishment of unmet needs after cervical cancer	In person group discussion PPI+RT
	Sharing personal experience of recovery after cervical cancer	One to one conversation PPI+SC
Recruitment for feasibility trial	PPI members provide feedback on recruitment poster	Email correspondence PPI+SC
	PPI members share study details via word of mouth or poster on online survivorship group	Email correspondence PPI+SC
Intervention design	PPI group to provide feedback on the feasibility of the intervention timeline	Group discussion between PPI + MR; use of questionnaire to elicit feedback
	PPI members to complete evaluation questionnaire and provide feedback on length and language use	One to one in person conversation PPI+SC
	PPI group to evaluate Intervention materials (diary, education and *Fitbit* guide)	Email correspondence PPI+SC
	PPI group to complete intervention launch session and provide feedback	Virtual group discussion PPI+SC
Programme delivery	PPI group to feedback on progress of intervention at mid-intervention delivery	Virtual group discussion PPI+SC
Dissemination of results	PPI group to feedback on appropriate strategies to disseminate results to the wider public (inc. patients and survivors)	Virtual group discussion PPI+SC

KEY: PPI= Patient and Public Involvement RT= Research Team; SC=Study Co-ordinator; TBC= to be confirmed

For peer review only

For peer review only - http://bmjopen.bmj.com/site/about/guidelines.xhtml

Participants and recruitment

Participants between the ages of 18 and 60 years who have undergone treatment for cervical cancer at least 6 months previously with curative intent (either surgery, chemoradiotherapy or both modalities), who do not meet the national PA guidelines (at least 150 minutes of moderate to vigorous intensity PA per week) [30], who can gain permission from their gynaecologist/ oncologist to take part and are proficient in speaking English will be recruited. The age range of 18 to 60 years was chosen to reflect the Chief Medical Officer's PA adult guidelines and to optimize engagement and adherence to a technology-based PA programme. Exclusion criteria are benign or pre-malignant (cervical interepithelial neoplasia) disease of the cervix; clinical/radiological evidence of disseminated malignancy; pregnancy or breastfeeding; performance status ≥3; co-morbidity that in the opinion of the patient's supervising gynaecologist/ oncologist that would preclude the patient from meeting the study requirements.

Initial data base screening will take place by the clinical gynaecological oncology team to identify candidates who are at an appropriate age and stage in their treatment journey. Potentially eligible participants will be approached through their clinical team, recruitment posters will be placed in the gynaecological and oncology outpatient departments at UHL and study details (overview of research, inclusion criteria and relevant contact details) will be posted on social media platforms. Local cervical cancer support groups will also be contacted and given information on the study to raise awareness of the research. Screening will take place via a telephone call with a member of the research team. The Scottish Physical Activity Questionnaire [31] will be used to determine PA levels. Participants will be asked how many minutes per week they spend being active at a moderate intensity in the context of the last 7 days. Consent procedures will take place either virtually via video/ telephone call or in person at UHL.

For peer review only - http://bmjopen.bmj.com/site/about/guidelines.xhtml

Sample Size

This study will aim to recruit approximately 30 participants. This sample size was chosen as it is a realistic target when considering cervical cancer incidence both in the U.K and at UHL where recruitment will take place over the given time period. In line with recommendations from the National Institute for Health Research (NIHR), a sample size of 30 is appropriate to answer the questions posed by a feasibility trial.[32] The feasibility of lifestyle and PA interventions in endometrial and breast cancer survivors have successfully employed similar sample sizes.[33,34]

Intervention

The aim of the multi-component intervention is to increase PA levels of the target population, specifically, individual and group-based walking over 12 consecutive weeks (Figure 1). Table 2 provides a description of each intervention component whilst Figure 2 outlines the Logic Model. Walking has been identified as a common PA preference among gynaecological cancer survivors[35], particularly for those who do not meet the recommended PA guidelines.[36] The intervention components are underpinned by social cognitive theory (SCT),[37] and are informed by the health belief model,[38] and theories of self-regulation.[28] Self-monitoring behaviour is strongly linked with successful PA behaviour change, particularly when combined with at least one other self-regulatory component.[39] The intervention includes education provision, problem solving and goal setting in relation to increasing PA levels after treatment for cervical cancer. Self-monitoring of PA behaviour will be facilitated by providing participants with an intervention diary to complete daily and weekly, fortnightly online health coaching via video or telephone call administered by the study co-ordinator and a consumer PA self-monitoring device to receive real time feedback on their activity levels and prompts to be more active and to review goals. Throughout, peer support and group walking amongst participants will be encouraged via a messaging platform which will allow participants to

For peer review only - http://bmjopen.bmj.com/site/about/guidelines.xhtml

maintain contact and organise group walking sessions. The study co-ordinator will suggest appropriate walk locations convenient to each group.

Table 2. Description of intervention components

**Intervention component**	**Description**
Intervention launch	**Education** (interactive session facilitated by the study co-ordinator, delivered virtually)<br>- What is meant by PA?<br>- What kinds of activities count as PA?<br>- What does moderate intensity PA feel like?<br>- What benefits are associated with PA?<br>- Knowledge of PA recommendations<br>- How many steps per day should you aim for?<br>**Barrier Identification**<br>- Is there anything that might stop you from being physically active?<br>- How can you overcome these challenges?<br>**Goal Setting**<br>- Introduction to SMART goals<br>- Using *Fitbit inspire* to set goals related to steps and intensity<br>*Each section divided by guided ‘stand up and stretch’ prompts
Self-monitoring of PA	- The PA monitor will provide participants with feedback on their activity levels via: number of steps taken by participants and the number of minutes that participants spend in a moderate intensity activity each day.
Prompts	- Prompts offered by PA monitor to encourage PA<br>- Prompts to update participant on their progress in reaching their daily goal.
Intervention diary (monitoring of well-being)	**Daily entries**<br>- Input daily step count as shown on Fitbit<br>- Rate mood out of 10<br>**Weekly entries**<br>- Rate the following symptoms on a traffic light system: energy levels, anxiety levels, body confidence, physical pain, bladder issues.
Health coaching	- Revision of previous goal(s) and the outcome (successful/ unsuccessful); attribute reasons for this outcome<br>- Identification of challenges and facilitators regarding previous goal<br>- Revision of steps, intensity and well-being over the previous two weeks<br>- Facilitated goal setting and identification of potential challenges for the following 2 weeks
Group messaging	- To be used as a platform to schedule group walks (organising a time and a place) with the aim of one group walk per week. Standardised communication prompts to be offered by the study co-ordinator.
Online community	- Participants will be encouraged to utilise the study specific community on the PA monitor application, accessed via smart phone where personal PA insights and

For peer review only - http://bmjopen.bmj.com/site/about/guidelines.xhtml

statistics can be shared with other participants.

Measures

All PA and health measures will be assessed at baseline, week-12 and 3- months after completion (week-24). Previous literature suggests that psychological measures may take longer to change than physical measures (e.g., menopausal symptoms or PA) and therefore to reduce participant burden, only these will be taken at week- 6 (table 3).

Table 3. PA and health outcomes, outcome measures and measurement time points

**Measures**	**Assessment tool**	**Baseline**	**Week 6**	**Week 12**	**Week 24**
Device- assessed PA	Accelerometer	x	x	x	x
Menopausal Symptoms	MRS	x	x	x	x
Quality of life	EORTC-QLQ-30	x		x	x
Depression and Anxiety	HADS	x		x	x
Fatigue	MSFI-SF	x		x	x
Walking Self- efficacy	Self- efficacy for Walking scale	x		x	x
Motivation for Exercise	BREQ-3	x		x	x
Enjoyment of PA	PACES	x		x	x

PA and health measures

An accelerometer (GENEActiv, Activinsights Ltd) and sleep log will be administered at all evaluation time points to measure PA. Participants will be asked to wear the accelerometer on their non-dominant wrist 24/7 for 8 days at each time point. Valid data will be at least 3 days >16 hours) of accelerometer data collected.(e.g. [40]) Device assessed PA will the primary research outcome measure of interest in the subsequent pilot study and definitive main trial.

For peer review only - http://bmjopen.bmj.com/site/about/guidelines.xhtml

Participants' belief in their ability to successfully complete incremental 5 to 50- minute periods of walking at a moderate/ brisk pace will be assessed via the self-efficacy for walking scale [41]. Motivation for PA will be assessed using the behavioural regulation in exercise questionnaire (BREQ-3).[42] This is a 23- item scale which assesses motivation on a self-determination continuum. Enjoyment of PA will also be assessed via the original 18- item PACES scale.[43]

In relation to well- being, menopausal symptoms, such as hot flushes and sweating will be investigated using the menopausal rating Scale (MRS).[44] Quality of life will be assessed using the EORTC QLQ- 30, which is commonly used in gynaecological cancer populations and includes 5 functional scales, 3 symptom scales, a global health scale and a quality of life scale. The Hospital Anxiety and Depression scale (HADS),[45] will be administered to measure symptoms of anxiety and depression. Fatigue will be assessed using the Multi-dimensional fatigue symptom inventory (MFSI-SF),[46] a measure which has been previously used to assess fatigue in a gynaecological cancer population.[23]

Evaluation related feasibility measures

Participant recruitment rates will be monitored by recording the number of identified potential participants, the number of those who express an interest in taking part, the number of those who are eligible and not eligible along with the reasons for ineligibility and the number who consent to participate. Retention and attrition rates will be monitored by recording the number of participants who withdraw from the study and the number who do not attend follow-up. Where possible, reasons for withdrawal will also be reported. The number of participants who complete the PA and health measures at each time point will be summarised.

Process evaluation

For peer review only - http://bmjopen.bmj.com/site/about/guidelines.xhtml

The process evaluation will be conducted to explore participant experiences of the study and intervention as well as compliance with the intervention components, to identify facilitators and barriers to increasing PA and any suggestions for improvement and refinement. Compliance with intervention components will be reviewed throughout the intervention. Data will be presented on launch attendance rates, the number of diary entries completed, number of daily step counts recorded to reflect compliance of wearing the PA monitor, number of health coaching sessions attended, the number of participants who post on the online forum and engage in group messaging, the number of participants who take part in group walks and the frequency of these.

Questionnaires will be administered to understand participant's experience of attending the intervention launch, of using the PA monitor and the group messaging. A semi- structured interview will also be conducted by the study co-ordinator to further explore perceptions of the intervention and the evaluation measures, perceived benefits of participation, maintenance of the intervention components and PA levels. Interview data will be analysed using Template Analysis,[47] employing a deductive coding framework to inform study refinement. To ensure a balanced insight and that subsequent meaningful refinement can be implemented, some transcripts will be coded by an independent researcher who did not contribute to the intervention design.

A researcher log will be completed by the study co-ordinator where a record of interactions with participants (e.g. email conversations/ telephone conversations) will be kept throughout the duration of the study. Feedback will be broadly divided into four categories: evaluation of intervention components; experience of evaluation measures; alterations to future intervention implementation; barriers to PA and reasons for discontinuation (where relevant).

Data collection

For peer review only - http://bmjopen.bmj.com/site/about/guidelines.xhtml

The accelerometer and the quantitative measures will be posted to participants. Process evaluation questionnaires will be completed online throughout the intervention. The evaluation interview will be offered after the week-24 time point to all participants (including those who have withdrawn) either in person or via video call.

Statistical Analysis

As this is a feasibility study, main analyses will utilise descriptive statistics. Data will be analysed using *IBM SPSS statistics 25*.

The feasibility of inclusion criteria

We have suggested that if an eligibility criterion accounts for 40% of ineligibility, it will be reported.

The feasibility of study recruitment and retention

A priori, we have defined a recruitment success rate as recruitment of 30 participants in the first 3- months or that 75% of those identified as eligible are recruited, and a successful retention rate at week-24 as 70%.

The feasibility of and compliance with intervention components

Attendance rates at the intervention launch, engagement with the online forum, frequency of wearing the PA monitor, frequency of group walking sessions, compliance with diary completion and attendance rates at health coaching sessions will all be summarised. The threshold of feasibility for these rates will be 70% of participants complying respectively.

The feasibility of the evaluation measures

Compliance with evaluation measures will be deemed successful if no more than 20% of participants fail to provide questionnaire and accelerometer data at both baseline and week-24 follow-up.

For peer review only - http://bmjopen.bmj.com/site/about/guidelines.xhtml

Physical activity data and questionnaire outcomes

Data from the accelerometer will be cleaned and processed in GGIR using *R programming* and analysed using *IBM SPSS statistics 25*. Means and standard deviation (SD) will be used to describe the PA from the accelerometer at each evaluation time point. Constructs measured via questionnaire will be analysed by standard scoring procedures. Questionnaire outcomes of interest will be reported on means (or medians where necessary) and SDs.

Ethics and dissemination

Ethical approval has been granted by the West of Scotland Research Ethics Committee 1 for this study. Results will primarily be used to refine intervention components and to inform a larger full-scale trial, if appropriate. Results from the study will be shared in peer reviewed journals, presented at international conferences and will be shared with the University Hospitals of Leicester and Leicester Hospital's charity.

For peer review only - http://bmjopen.bmj.com/site/about/guidelines.xhtml

DISCUSSION

This article describes the protocol for a study testing the feasibility and acceptability of conducting and evaluating of a multi- component PA intervention following treatment for cervical cancer. Regular PA has been shown to have physical and psychological benefits and to be safe after cancer .[48] PA can also alleviate a range of symptoms experienced as a result of cancer treatment .[23] PA levels appear to be low following cervical cancer [21] and this may be partly due to the lack of a specific intervention that takes into consideration the particular side-effects that can occur, in particular pelvic pain and incontinence, that may not be such an issue in other cancer types.

The study protocol details a 12-week intervention programme to promote walking and PA, with evaluation measures taken at baseline, 6- weeks, 12 weeks and 24 weeks. The process evaluation is a mix of questionnaires and a qualitative interview. The intervention was designed using the intervention mapping protocol.[28] Development followed a systematic 6-step framework, which prioritises input from the target population as well as integrating behaviour change theories (BCTs) and constructs to underpin change within the intervention, informed by relevant literature.[49] Interventions underpinned by relevant BCTs have been found to be more successful in implementing change and in identifying more specific evaluation criteria.[48]

The current PA guidelines offered by the Government Chief Medical Officers do not specify recommendations for cervical cancer survivors.[30] However, using a development framework that prioritises insights and experiences from the target population is a great strength of this

For peer review only - http://bmjopen.bmj.com/site/about/guidelines.xhtml

intervention, as it allowed direct and effective tailoring of the intervention components despite specific recommendations not being available. Hence, the aerobic PA recommendations were deemed an appropriate target for women treated for cervical cancer.[29] Additionally, the criterion to include women who are at least 6- months post treatment was also a decision informed by the need's assessment, with the time between 3 and 9 months post treatment being identified as critical for intervention and for positive change.[50] A limitation is that the protocol does not incorporate strength training, and therefore does not reflect the current PA guidelines for after cancer. However, as PA levels are generally low following cervical cancer treatment, it seemed appropriate to promote one PA modality which was realistically achievable (i.e. walking) to enhance adherence to the intervention.

This study will add to the current debate regarding the role of technology in promoting PA after cancer. The intervention utilises a PA monitor and its associated online community and a messaging platform. Previous research supports the use of such e-health technologies in combination with health coaching over a shorter duration (4-week programme).[51] However, it has been suggested that further investigation is needed to test the acceptability of such technologies in more diverse populations,[52] to explore optimal intervention length and to question the maintenance of PA behaviour change.[51] Although a limitation of this protocol is that statistically relevant changes in PA between time points will not be reported, trends in PA levels will provide data inferring the feasibility of promoting and maintaining PA behaviours. The process evaluation will also provide valuable insight regarding the acceptability of the components.

For peer review only

For peer review only - http://bmjopen.bmj.com/site/about/guidelines.xhtml

CONCLUSION

We described the protocol of a study which tests the feasibility and acceptability of delivering and evaluating a multi- component physical activity intervention for women following treatment for cervical cancer. A process evaluation plan will provide insight into whether the intervention components and evaluation measures are accepted by participants. Findings from the study will inform intervention refinement in preparation for a full-scale pilot trial.

For peer review only - http://bmjopen.bmj.com/site/about/guidelines.xhtml

Contributorship statement

All authors contributed to the study conception and design and protocol preparation. The first draft of the manuscript was written by NM and ELM. CE, FM and HJM significantly revised the manuscript. All authors read and approved the final manuscript.

Competing Interest statement

ELM has received research grants from Intuitive Surgical and Hope Against Cancer for unrelated work. ELM has received lecture fees for GlaxoSmithKlein and has served on the clinical advisory boards for Inivata and GlaxoSmithklein.

Funding Statement

This research is supported by the National Institute for Health Research (NIHR) Leicester Biomedical Research Centre, which is a partnership between University Hospitals of Leicester NHS Trust, Loughborough University and the University of Leicester, UK. Support. Funding was provided by the Economic, Social and Research Council (ES/P000711/1), Loughborough university (ES/P000711/1), and the Leicester Hospital's charity, University Hospitals of Leicester (Q843).

Acknowledgements

The authors are very grateful to the members of the Patient and Public Involvement group for their contributions and insights throughout the intervention development stages.

For peer review only - http://bmjopen.bmj.com/site/about/guidelines.xhtml

REFERENCES

1 CRUK U. Cervical cancer statistics. 2017.

2 Vale CL, Tierney JF, Davidson SE, *et al.* Substantial Improvement in UK Cervical Cancer Survival with Chemoradiotherapy: Results of a Royal College of Radiologists' Audit. *Clin Oncol* Published Online First: 2010. doi:10.1016/j.clon.2010.06.002

3 Moss EL, Taneja S, Munir F, *et al.* Iatrogenic Menopause After Treatment for Cervical Cancer. *Clin Oncol* 2016;**28**:766–75. doi:10.1016/j.clon.2016.08.016

4 Public Health England. Cervical cancer screening: invasive cervical cancer audit 2013 to 2016. 2019.https://www.gov.uk/government/publications/cervical-screening-invasive-cervical-cancer-audit-2013-to-2016/executive-summary#staging-information

5 Bergmark K, Åvall-Lundqvist E, Dickman PW, *et al.* Patient-rating of distressful symptoms after treatment for early cervical cancer. *Acta Obstet Gynecol Scand* Published Online First: 2002. doi:10.1034/j.1600-0412.2002.810512.x

6 Pisarska M, Sajdak S. Lower urinary tract function after postoperative radiotherapy in the treatment of cervical cancer. *Eur J Gynaecol Oncol* 2003.

7 Sood AK, Nygaard I, Shahin MS, *et al.* Anorectal dysfunction after surgical treatment for cervical cancer. *J Am Coll Surg* Published Online First: 2002. doi:10.1016/S1072-7515(02)01311-X

8 Butler-Manuel SA, Summerville K, Ford A, *et al.* Self-assessment of morbidity following radical hysterectomy for cervical cancer. *J Obstet Gynaecol (Lahore)* Published Online First: 1999. doi:10.1080/01443619965552

9 Jensen PT, Groenvold M, Klee MC, *et al.* Longitudinal study of sexual function and vaginal changes after radiotherapy for cervical cancer. *Int J Radiat Oncol Biol Phys* Published Online First: 2003. doi:10.1016/S0360-3016(03)00362-6

10 Elghamrawi KA, Haggag MH, Habib EE. Treatment complications among long-term survivors of cervical cancer: Treated by surgery or radiotherapy. Oncol. Rev. 2007. doi:10.1007/s12156-011-0093-0

11 Schover LR, Fife M, Gershenson DM. Sexual dysfunction and treatment for early stage cervical cancer. *Cancer* Published Online First: 1989. doi:10.1002/1097-0142(19890101)63:1<204::AID-CNCR2820630133>3.0.CO;2-U

12 Le Borgne G, Mercier M, Woronoff AS, *et al.* Quality of life in long-term cervical cancer survivors: A population-based study. *Gynecol Oncol* 2013;**129**:222–8. doi:10.1016/j.ygyno.2012.12.033

13 Derks M, Van Lonkhuijzen LRCW, Bakker RM, *et al.* Long-Term Morbidity and

For peer review only - http://bmjopen.bmj.com/site/about/guidelines.xhtml

Quality of Life in Cervical Cancer Survivors: A Multicenter Comparison between Surgery and Radiotherapy as Primary Treatment. *Int J Gynecol Cancer* Published Online First: 2017. doi:10.1097/IGC.0000000000000880

14 Kirchhoff AC, Yi J, Wright J, *et al.* Marriage and divorce among young adult cancer survivors. *J Cancer Surviv* Published Online First: 2012. doi:10.1007/s11764-012-0238-6

15 Sekse RJT, Raaheim M, Blaaka G, *et al.* Life beyond cancer: Women's experiences 5-years after treatment for gynaecological cancer. *Scand J Caring Sci* 2010;**24**:799–807. doi:10.1111/j.1471-6712.2010.00778.x

16 Faller H, Brähler E, Härter M, *et al.* Unmet needs for information and psychosocial support in relation to quality of life and emotional distress: A comparison between gynecological and breast cancer patients. *Patient Educ Couns* 2017;**100**:1934–42. doi:10.1016/j.pec.2017.05.031

17 Caspersen CJ, Powell KE, Christenson GM. Physical activity, exercise, and physical fitness: definitions and distinctions for health-related research. *Pblic Heal Rep* 1985;**100**:126–31. doi:10.1093/nq/s9-IX.228.365-f

18 Buffart LM, Kalter J, Sweegers MG, *et al.* Effects and moderators of exercise on quality of life and physical function in patients with cancer: An individual patient data meta-analysis of 34 RCTs. *Cancer Treat Rev* 2017;**52**:91–104. doi:10.1016/j.ctrv.2016.11.010

19 Burke S, Wurz A, Bradshaw A, *et al.* Physical activity and quality of life in cancer survivors: A meta-synthesis of qualitative research. Cancers (Basel). 2017. doi:10.3390/cancers9050053

20 Fleming S, Jones T, Janda M, *et al.* Physical activity trajectories following gynecological cancer: results from a prospective, longitudinal cohort study. *Int J Gynecol Cancer* Published Online First: 2020. doi:10.1136/ijgc-2020-001543

21 Thraen-Borowski KM, Gennuso KP, Cadmus-Bertram L. Accelerometer-derived physical activity and sedentary time by cancer type in the United States. *PLoS One* Published Online First: 2017. doi:10.1371/journal.pone.0182554

22 Campbell KL, Winters-Stone KM, Wiskemann J, *et al.* Exercise Guidelines for Cancer Survivors: Consensus Statement from International Multidisciplinary Roundtable. *Med Sci Sports Exerc* 2019;**51**:2375–90. doi:10.1249/MSS.0000000000002116

23 Donnelly CM, Blaney JM, Lowe-Strong A, *et al.* A randomised controlled trial testing the feasibility and efficacy of a physical activity behavioural change intervention in managing fatigue with gynaecological cancer survivors. *Gynecol Oncol* 2011;**122**:618–24. doi:10.1016/j.ygyno.2011.05.029

24 Donnelly CM, Lowe-Strong A, Rankin JP, *et al.* A focus group study exploring gynecological cancer survivors' experiences and perceptions of participating in a RCT testing the efficacy of a home-based physical activity intervention. *Support Care Cancer* 2013;**21**:1697–170. doi:10.1007/s00520-012-1716-0

25 Brown JC, John GM, Segal S, *et al.* Physical activity and lower limb lymphedema among uterine cancer survivors. *Med Sci Sports Exerc* 2013;**45**:2091. doi:10.1249/MSS.0b013e318299afd4

For peer review only - http://bmjopen.bmj.com/site/about/guidelines.xhtml

26 Rossi A, Garber CE, Ortiz M, *et al.* Feasibility of a physical activity intervention for obese, socioculturally diverse endometrial cancer survivors. *Gynecol Oncol* 2016;**142**:304–10. doi:10.1016/j.ygyno.2016.05.034

27 Rossi A, Frechette L, Miller D, *et al.* Acceptability and feasibility of a Fitbit physical activity monitor for endometrial cancer survivors. *Gynecol Oncol* 2018;**149**:470–5. doi:10.1016/j.ygyno.2018.04.560

28 Bartholomew, Eldredge L Highfield L, Hartman M, Mullen P, *et al.* Using Intervention Mapping to Adapt Evidence-Based Interventions. In: *Planning health promotion programs : an intervention mapping approach*. 2016.

29 Millet N, McDermott HJ, Moss EL, *et al.* Increasing physical activity levels following treatment for cervical cancer: an intervention mapping approach. *J Cancer Surviv* Published Online First: 2021. doi:10.1007/s11764-021-01058-y

30 Davies DSC, Atherton F, McBride M, *et al.* UK Chief Medical Officers' Physical Activity Guidelines. 2019.

31 Lowther M, Mutrie N, Loughlan C, *et al.* Development of a Scottish physical activity questionnaire: A tool for use in physical activity interventions. *Br J Sports Med* Published Online First: 1999. doi:10.1136/bjsm.33.4.244

32 Lancaster GA, Dodd S, Williamson PR. Design and analysis of pilot studies: Recommendations for good practice. J. Eval. Clin. Pract. 2004. doi:10.1111/j..2002.384.doc.x

33 Rossi A, Frechette L, Miller D, *et al.* Acceptability and feasibility of a Fitbit physical activity monitor for endometrial cancer survivors. *Gynecol Oncol* 2018;**149**:470–5. doi:10.1016/j.ygyno.2018.04.560

34 Pullen T, Sharp P, Bottorff JL, *et al.* Acceptability and satisfaction of project MOVE: A pragmatic feasibility trial aimed at increasing physical activity in female breast cancer survivors. *Psychooncology* Published Online First: 2018. doi:10.1002/pon.4662

35 Farrokhzadi L, Dhillon HM, Goumas C, *et al.* Physical Activity Correlates, Barriers, and Preferences for Women with Gynecological Cancer. In: *International Journal of Gynecological Cancer*. 2016. doi:10.1097/IGC.0000000000000790

36 Stevinson C, Tonkin K, Capstick V, *et al.* A Population-Based Study of the Determinants of Physical Activity in Ovarian Cancer Survivors. *J Phys Act Heal* 2009;**6**:339–46. doi:10.1123/jpah.6.3.339

37 Bandura A. *Social foundations of thought and action : a social cognitive theory / Albert Bandura.* 1986.

38 Janz K, Becker MH. The Health Belief Model : A Decade Later. 1984;:1–47.

39 Michie S, Abraham C, Whittington C, *et al.* Effective Techniques in Healthy Eating and Physical Activity Interventions: A Meta-Regression. *Heal Psychol* Published Online First: 2009. doi:10.1037/a0016136

40 Yates T, Griffin S, Bodicoat DH, *et al.* PRomotion Of Physical activity through structured Education with differing Levels of ongoing Support for people at high risk of type 2 diabetes (PROPELS): Study protocol for a randomized controlled trial. *Trials* Published Online First: 2015. doi:10.1186/s13063-015-0813-z

For peer review only - http://bmjopen.bmj.com/site/about/guidelines.xhtml

41 McAuley E, Blissmer B, Katula J, *et al.* Exercise environment, self-efficacy, and affective responses to acute exercise in older adults. *Psychol Heal* Published Online First: 2000. doi:10.1080/08870440008401997

42 Markland D, Tobin V. A modification to the behavioural regulation in exercise questionnaire to include an assessment of amotivation. *J Sport Exerc Psychol* Published Online First: 2004. doi:10.1123/jsep.26.2.191

43 Kendzierski D, DeCarlo KJ. Physical Activity Enjoyment Scale: Two Validation Studies. *J Sport Exerc Psychol* Published Online First: 2016. doi:10.1123/jsep.13.1.50

44 Hauser GA, Huber IC, Keller PJ, *et al.* EVALUATION DER KLIMAKTERISCHEN BESCHWERDEN (MENOPAUSE RATING SCALE [MRS]). Zentralbl. Gynakol. 1994.

45 Zigmond, Anthony S, Snaith, Philip R. The Hospital Anxiety and Depression Scale. *Acta Psychiatr Scand* 1983;**67**:361–70.

46 Stein KD, Jacobsen PB, Blanchard CM, *et al.* Further validation of the multidimensional fatigue symptom inventory-short form. *J Pain Symptom Manage* Published Online First: 2004. doi:10.1016/j.jpainsymman.2003.06.003

47 King N. Doing Template Analysis. In: *Qualitative Organizational Research: Core Methods and Current Challenges*. 2017. doi:10.4135/9781526435620.n24

48 Turner RR, Steed L, Quirk H, *et al.* Interventions for promoting habitual exercise in people living with and beyond cancer. *Cochrane Database Syst Rev* Published Online First: 2018. doi:10.1002/14651858.CD010192.pub3

49 Hirschey R, Bryant AL, Macek C, *et al.* Predicting physical activity among cancer survivors: Meta-analytic path modeling of longitudinal studies. *Heal Psychol* Published Online First: 2020. doi:10.1037/hea0000845

50 Sabiston CM, Brunet J, Vallance JK, *et al.* Prospective Examination of Objectively Assessed Physical Activity and Sedentary Time after Breast Cancer Treatment : Sitting on the Crest of the Teachable Moment. 2014;**23**:1324–31. doi:10.1158/1055-9965.EPI-13-1179

51 Gell NM, Grover KW, Humble M, *et al.* Efficacy, feasibility, and acceptability of a novel technology-based intervention to support physical activity in cancer survivors. *Support Care Cancer* Published Online First: 2017. doi:10.1007/s00520-016-3523-5

52 Haberlin C, O'Dwyer T, Mockler D, *et al.* The use of eHealth to promote physical activity in cancer survivors: a systematic review. Support. Care Cancer. 2018. doi:10.1007/s00520-018-4305-z

Protocol amendments

Any modifications to the protocol will be approved by the West of Scotland Research Ethics Committee 1 prior to implementation and notified to the health authorities in accordance with local regulations.

For peer review only - http://bmjopen.bmj.com/site/about/guidelines.xhtml

Ethical approvals

Ethical approval was given by the West of Scotland Research Ethics Committee (20/WS/0062).

List of Figures

Figure 1. Intervention Programme Flowchart

*Figure 2***.** Logic Model for the ACCEPTANCE trial

For peer review only

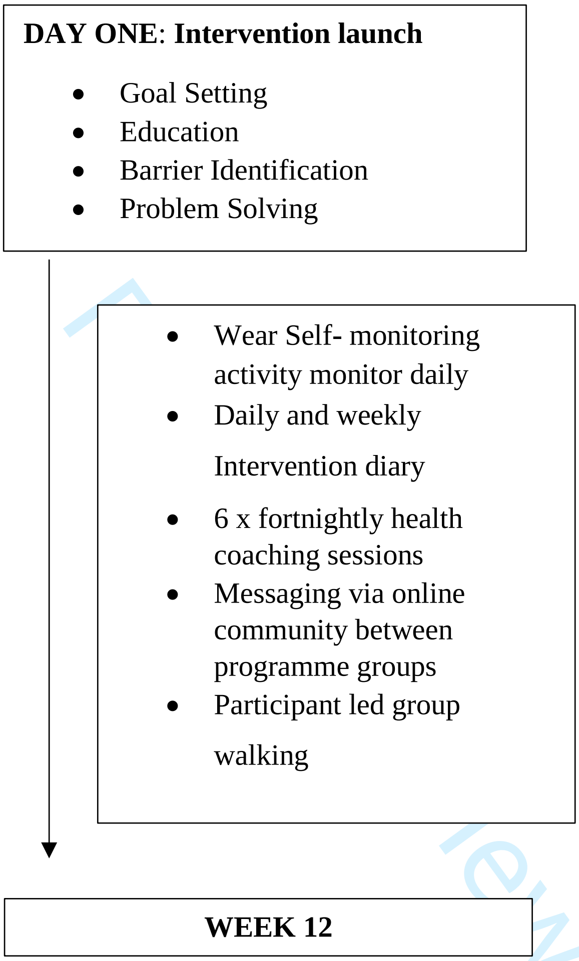

Figure 1. Intervention programme flowchart

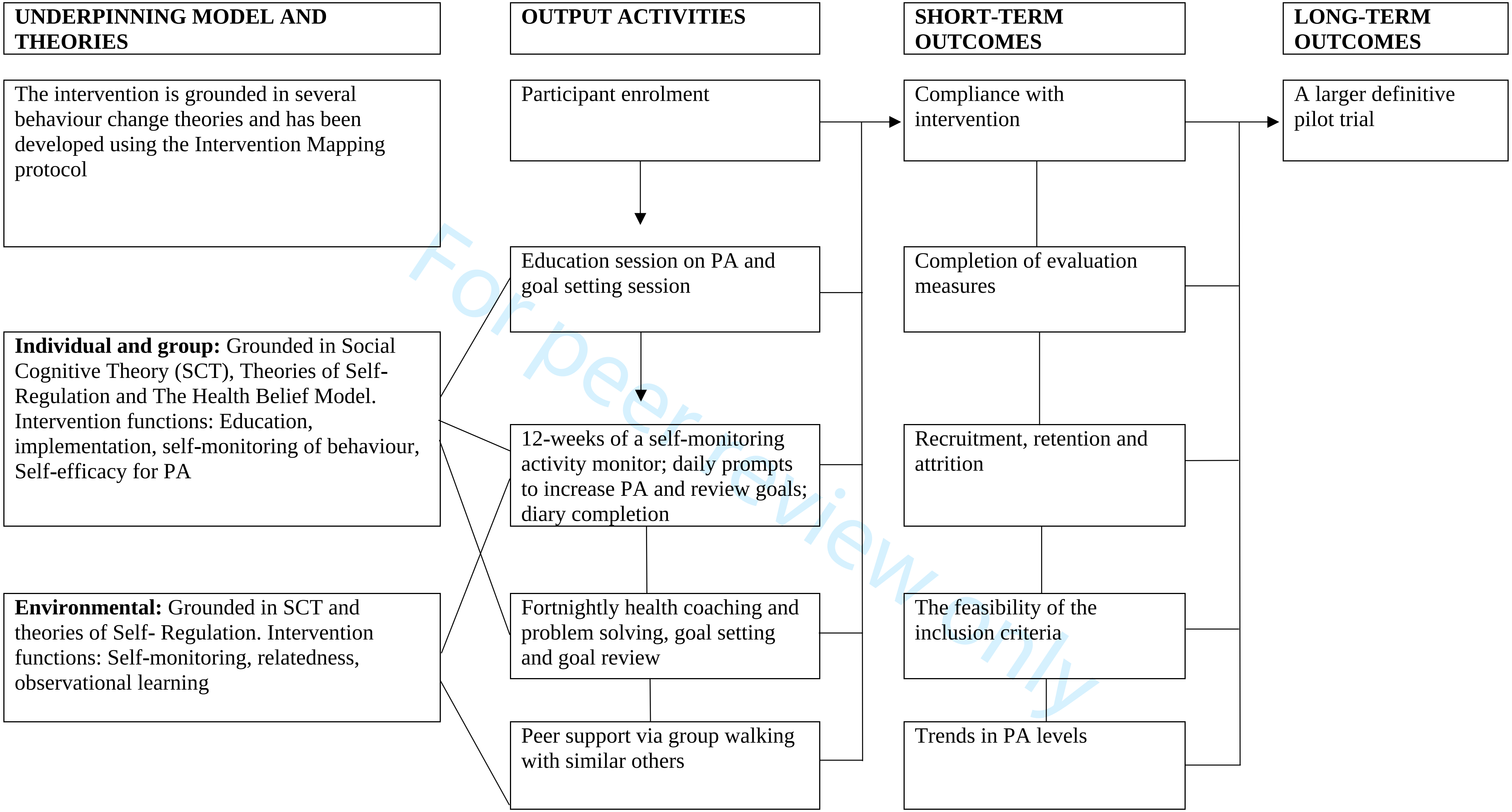

Figure 2. The logic model for the ACCEPTANCE study

For peer review only
